# Genomic tools for post-elimination measles molecular epidemiology using Canadian surveillance data from 2018–2020

**DOI:** 10.3389/fmicb.2024.1475144

**Published:** 2024-11-19

**Authors:** Joanne Hiebert, Vanessa Zubach, Helene Schulz, Alberto Severini

**Affiliations:** ^1^Measles, Mumps and Rubella Unit, National Microbiology Laboratory Branch, Public Health Agency of Canada, JC Wilt Infectious Diseases Research Centre, Winnipeg, MB, Canada; ^2^Department of Medical Microbiology and Infectious Diseases, Faculty of Health Sciences, University of Manitoba, Winnipeg, MB, Canada

**Keywords:** measles, surveillance, elimination, genome, epidemiology

## Abstract

**Introduction:**

Measles is caused by the highly infectious measles virus, MeV, for which there is an effective vaccine. Monitoring of progress of measles elimination requires enhanced surveillance and tracking of MeV strains, including documenting the absence of an endemically circulating strain. Due to a reduction in the number of circulating genotypes, additional sequence information, beyond the standardized 450 nucleotide window of the nucleoprotein (N450), is required to corroborate the information from epidemiological investigations and, ideally, fill in gaps in the surveillance data.

**Methods:**

This study applies MeV sequencing tools, namely the N450, the non-coding region between the matrix and fusion genes (MF-NCR), and the complete coding sequence of the genome (WGS-t), to clinical specimens obtained from cases occurring over a three-year time period in Canada. This data was systematically analyzed, including with Bayesian evolutionary analysis by sampling trees (BEAST) of the WGS-t.

**Results and discussion:**

Of the 143 reported cases, N450, MF-NCR, and WGS-t sequences were obtained from 101, 81, and 75 cases, respectively. The BEAST analysis confirmed that the two most frequently detected lineages (B3 named strain MVi/Marikina City.PHL/10.18 and D8 named strain MVs/Gir Somnath.IND/42.16) were the result of repeated importations. Of the 16 outbreaks occurring during the study period, the analysis conclusively corroborated the epidemiological information for 13. BEAST analysis of the WGS-t convincingly demonstrated the expansion of two outbreaks by the inclusion of additional contemporary cases for which the epidemiological investigation had been unable to identify links. Furthermore, the analysis revealed the existence of three additional unrecognized outbreaks among the cases categorized as unknown source. One outbreak was without WGS-t and could not be resolved.

**Conclusion:**

Measles WGS-t data corroborated and expanded upon the outbreak analysis from traditional epidemiological investigations of measles outbreaks. However, both are needed for fulsome investigations in elimination settings.

## Introduction

Measles virus (MeV), an enveloped paramyxovirus with a negative-sense, single-stranded RNA genome typically 15,894 nucleotides in length, causes the highly infectious measles disease characterized by fever and rash (Plemper and Lamb, 2021; Phan et al., 2018). It is preventable through the use of live attenuated vaccines, which were developed in the 1960s (Tulchinsky, 2018). As humans are the only host and there is an effective vaccine available, the World Health Organization (WHO) has targeted measles for eradication (Rota et al., 2016). Every global WHO region has established elimination goals, which have been achieved in several countries including Canada (Minta et al., 2023; King et al., 2004). Monitoring of progress toward these goals requires enhanced, case-based surveillance of measles cases and tracking of MeV strains (World Health Organization, 2013; Brown et al., 2019). One of the essential criteria for verifying the elimination of endemic measles transmission is demonstrating the absence of an endemic viral strain (World Health Organization, 2013; Sniadack et al., 2017). As MeV is serologically monotypic, differentiation is done by WHO-standardized genotyping, which is based on the sequence of the carboxy-terminal 450 nucleotides of the nucleoprotein open reading frame (the N450) (World Health Organization, 1998; World Health Organization, 2012; Mulders et al., 2015). While 24 genotypes were established, many have become extinct over the years, with only four genotypes reported to WHO’s measles sequence database, MeaNS, in 2018 and only three in 2020 (Brown et al., 2019; Mulders et al., 2015; Dixon et al., 2021). As a result, identification of the MeV genotype is no longer sufficient for monitoring transmission; instead, the WHO global measles and rubella laboratory network (GMRLN) has developed a scheme that labels exact N450 sequences with distinct sequence identifiers (DSIds), some of which are designated as so-called “named strains” (Williams et al., 2022). Furthermore, the MeV genome is very stable and the same DSId can be detected in multiple geographic areas simultaneously for months or years. Indeed, the designation of a DSId as a “named strain” requires that the DSId be detected in at least 50 cases in more than two countries and over at least two years (Williams et al., 2022). According to a 2019 report on circulating MeV strains, two named strains were detected in all six WHO global regions between 2016 and 2018 (Brown et al., 2019). This same report documented a reduction in both the number of MeV genotypes and DSIds, even though the number of sequence submissions went up over the time period (Brown et al., 2019). Therefore, there is a need to gather additional MeV sequence information from cases of measles to better document and establish chains of transmission. The GMRLN has proposed the addition of either the non-coding sequence between the matrix (M) and fusion (F) genes (dubbed the MF-NCR) or the complete coding sequence of the genome (dubbed WGS-t) as additional sources of sequence information, particularly in countries that have eliminated endemic measles circulation (World Health Organization, 2017).

Methods to obtain WGS-t from MeV cases necessarily require techniques to amplify or enrich the template viral sequence, which otherwise represents a very small portion of the overall sequence milieu. MeV sequence enrichment methods typically include amplification either by isolation in cell culture or RT-PCR amplification prior to sequencing (Gardy et al., 2015; Penedos et al., 2015; Vaidya et al., 2020; Probert et al., 2021; Jaafar et al., 2021; Song et al., 2022; Penedos et al., 2022; Cui et al., 2022; Bucris et al., 2024). However, these techniques come with the theoretical risk of introducing mutations into the sequence, which is problematic when unrelated cases may deviate by only a single nucleotide (Masters et al., 2023). We have previously established a next-generation sequencing (NGS) method for sequencing directly from clinical specimens using MeV template enrichment by hybridization probes (Schulz et al., 2022), an approach that has been used in only one study of MeV outbreaks (Masters et al., 2023). In this study, we sought to determine the feasibility of applying MF-NCR sequencing and WGS by NGS to MeV clinical specimens obtained from measles surveillance in Canada. In addition, we sought to explore the application of molecular epidemiology tools, including Bayesian approaches to phylogenetic analysis, to determine to what extent it can be used to complement the traditional measles case investigation. While Bayesian evolutionary analysis by sampling trees (BEAST) has been applied in other MeV WGS-t studies (Penedos et al., 2015; Song et al., 2022; Penedos et al., 2022), it has not previously been used to systematically interrogate the data to confirm epidemiological information and explore connections between phylogenetic clusters. Our dataset, consisting of MeV sequence data from 16 well-defined outbreaks, numerous instances of single imported MeV cases, as well as cases of unknown source, allowed the unique ability to analyze inter- and intra-cluster relatedness. We show that MeV WGS-t sequences can be successfully obtained from most clinical specimens, especially those with a CP/Ct value <27, and that phylogenetic analysis of MF-NCR and WGS-t sequences improves the resolution of the molecular epidemiology. Finally, we demonstrate that BEAST analysis corroborates the information obtained from traditional epidemiological investigations and provides a means to resolve cases of unknown source.

## Materials and methods

### Surveillance data, clinical specimens, and RNA extraction

Confirmed cases of measles are reported to the Public Health Agency of Canada’s (PHAC) Canadian Measles and Rubella Surveillance System (CMRSS), which collects detailed information on all cases (Public Health Agency of Canada, 2024). The information used in this study included onset dates, travel history, and epidemiologically defined links to other cases (outbreaks). Outbreaks are defined as two or more linked cases. The sources of exposure were classified as outside Canada (imported), within Canada and linked to an imported case (import-related), or unknown source/sporadic and were defined by the reporting province or territory (Coulby et al., 2020; Coulby et al., 2021).

All MeV genotyping in Canada is routinely performed at PHAC’s National Microbiology Laboratory (NML). Clinical specimens (in descending order of frequency: nasopharyngeal/throat swab, urine, nasopharyngeal aspirate, buccal swab, or unknown type) that were collected from confirmed cases of measles, with rash onset from the first week of 2018 to the last week of 2020 and RT-PCR positive for MeV, were included in this study (*n* = 101). Total nucleic acids were extracted using the MagNA Pure 96 DNA and Viral NA large volume kit on the MagNA pure 96 (Roche Diagnostics Corp., Indianapolis, IN, USA) or with the QIAamp Viral RNA Mini Kit (Qiagen), following manufacturer’s instructions. Extracted RNA was stored at −80°C. Real-time RT-PCR at the NML was performed as previously described (Zubach et al., 2022).

Analyses of specimen type and real-time RT-PCR results in comparison to sequencing success was performed in Microsoft Excel for Microsoft 365.

### RT-PCR amplification and sanger sequencing

RT-PCR amplification of the WHO standardized region of the nucleoprotein gene (the N450), Sanger sequencing (both using amplification primers MVN1109 and MVN1698R, and a sequencing primer MVN1231) and genotype assignments were done as previously described (Thomas et al., 2017). For the amplification of the non-coding region between the matrix (M) and fusion (F) open reading frames (ORF) (the MF-NCR), the first cDNA was generated using the Superscript VILO Synthesis kit (Invitrogen 11,754–250) following manufacturer’s instructions. The MF-NCR was PCR amplified from the cDNA using primer pair 4200F (GGC ACC AGT CTT CAC ATT AG) and 5601R (CGA GTC ATA ACT TTG TAG CTT GC), with a final primer concentration of 0.3 μM, and the Takara PrimerSTAR GXL DNA Polymerase (R050A) kit. Templates were initially denatured for 1 min at 98°C followed by 40 cycles of denaturing for 10s at 98°C, annealing for 15 s at 60°C, and extension for 90s at 68°C. The products were visualized on the QIAxcel capillary electrophoresis system (Qiagen) for the presence of a 1,401 bp amplicon, then prepared for sequencing using Exo-SAP-IT Express PCR Product Cleanup (Applied Biosystems 75001.40UL).

All sanger sequencing was performed by the NML’s in-house genomics Core and used the amplification primers. For sequencing of the MF-NCR, primers 4609F (AAC ACA AGG CCA CCA CCA G), 5126F (CAC ACA CGA CCA CGG CAA CC), 4712R (GGG ATG CGG TTG GTG GTC T), 4826R (GAC CGA GGT GAC CCA AC), and 5056R (CCC CCC GTC TTG GAY TGT CG) were used in addition to the amplification primers.

### Next-generation sequencing (Illumina) library preparation

Reverse Transcription and Next-Generation Sequencing (NGS) was carried out as previously described using enrichment with MeV specific probes (Schulz et al., 2022). First and second strand cDNA was synthesized; fragmentation then took place using the Covaris E220 (Woburn MA USA), followed by size selection using Agencourt AMPure XP beads (Beckman Coulter, A63880). The library preparation continued with the Illumina TruSeq DNA Nano LP (cat# 20016328), following manufacturer’s instructions. MeV-specific custom biotinylated lockdown DNA probes were sourced from Integrated DNA Technologies (Coralville, IA, USA); these probes were hybridized to MeV libraries and captured with Dynabeads™ M270-streptavidin magnetic beads (Thermofisher Scientific). MeV libraries bound to the streptavidin beads were subject to 12 cycles of PCR amplification using the universal Illumina primers that recognize the ligated adapter sequence of the libraries and enzymes from the Illumina TruSeq DNA Nano LP kit. The post-capture PCR fragments were purified with Agencourt AMPure XP beads (Beckman Coulter) and sequenced without further size selection on the MiSeq instrument (Illumina) by the NML’s in-house Genomics Core.

### Reference strains for data analysis

For use as reference sequences for read mapping during sequence and phylogenetic analysis, 34 MeV sequences were downloaded from GenBank (Supplementary Table S3).

### Generation of consensus sequences

Consensus sequences of Sanger sequencing data were generated with SeqManPro version 11 software (DNASTAR Lasergene, WI). Reference-mapped post-assembled reads were trimmed manually, removing primer sequence derived read-ends, to yield high quality consensus sequences, which were saved in “.fasta” file format. N450 sequences were trimmed to the WHO standardized window, and the MF-NCR sequences were trimmed to include the stop codon of the M ORF and the start codon of the F ORF and were 1,018 nucleotides in length.

NGS data quality was checked with FastQC.^1^ Geneious Prime version 2022.1.1 was used to quality trim the data and map it to a genotype specific GenBank reference strain (Supplementary Table S3) using the Geneious mapper at medium sensitivity. Consensus sequences were trimmed to exclude the termini (WGS-t), specifically starting from the start codon of the N ORF and ending at the stop codon of the L ORF. For most samples the consensus sequences had at least 10-fold coverage and the threshold was set to Highest Quality (60%): bases matching at least 60% of total adjusted chromatogram quality were saved in “.fasta” file format. The WGS-t sequences in this study were 15,678 nucleotides in length.

Consensus sequences for all loci were named according to the WHO standardized method (World Health Organization, 2012). The epidemiological week (starting with the first Monday in January) was based on the WHO week numbering system, which resulted in slightly different weeks assigned than what appeared in the previously published surveillance reports (Coulby et al., 2020; Coulby et al., 2021). Distinct sequence identifiers (DSIds) were assigned to the N450 consensus sequences using the WHO MeV sequence database, MeaNS (Mulders et al., 2015; Williams et al., 2022).

### Phylogenetic and pairwise distance analysis

Consensus sequences were aligned using MEGA version X Clustal W alignment with default settings (Kumar et al., 2018). Phylogenetic trees of the aligned sequences were inferred by the Maximum Likelihood method, with 1,000 bootstrap replicates. Final trees were annotated with meta-data in the iTOL (Interactive Tree of Life) online annotator, version 6.5.8, available at: https://itol.embl.de (Letunic and Bork, 2021). Pairwise distances were calculated in MEGA version X (Kumar et al., 2018) using the Maximum Composite Likelihood model with default settings (Tamura et al., 2004). Plots and statistical analysis (Mann–Whitney test) of this data were made in GraphPad Prism version 10.1.2.

### Similarity plot (SimPlot) analysis

The nucleotide sequence similarity of the consensus sequences generated were plotted against a query sequence (GenBank AF266288 Genotype A) using SimPlot software (version 3.5.1) (Lole et al., 1999). The sequences were plotted using a 20-base step size using a moving window of 200 bases.

### Bayesian evolutionary analysis by sampling trees (BEAST)

BEAST2 v2.6.7 (Bouckaert et al., 2014) was used to construct time-scaled, maximum clade credibility (MCC) phylogenetic trees and obtain molecular clock rates for the MF-NCR region and WGS. Sequences deposited to GenBank from international sources, with an N450 sequence identical to the WHO named strain MVs/Gir Somnath.IND/42.16, were added to the alignment (Supplementary Table S3). Prior to a BEAST2 experiment, alignments from MEGA X were exported to a Nexus file and brought into BEAUTi v2.5. In BEAUTi, XML files were generated using the BEAST Model test, a strict clock, and either the Coalescent Constant Population or Coalescent Exponential Population tree prior model (specified in Supplementary Table S2). For each analysis, BEAST was run five times from different seeds with the same set of conditions. BEAST output logs were analyzed using Tracer v1.7.2 (Rambaut et al., 2018). Combined logs reached a minimum value of 1,000 Effective Sample Size (ESS) after removal of 10% burn-in and were converging. The log and tree files were combined using LogCombiner v2.6.7 for further analysis. TreeAnnotator v2.6.6 was used to summarize the data from a sample of trees into a single tree, which was viewed in FigTree v1.4.4.^2^ Final trees were annotated with meta-data in the iTOL (Interactive Tree of Life) online annotator, version 6.5.8 (Letunic and Bork, 2021).

### Data availability

Measles case information and sequences have been deposited into the MeaNS database. Complete genome sequences (minus termini) have been deposited into GenBank with the accession numbers OQ096264 to OQ096339. WGS-t raw read data have been submitted to SRA under BioProject PRJNA1017431. A line listing of accession numbers is available in Supplementary Table S1.

## Results and discussion

### Whole genome sequences can be obtained from most clinical specimens over a wide range of measles RT-PCR positivity with high accuracy

**Figure 1:**
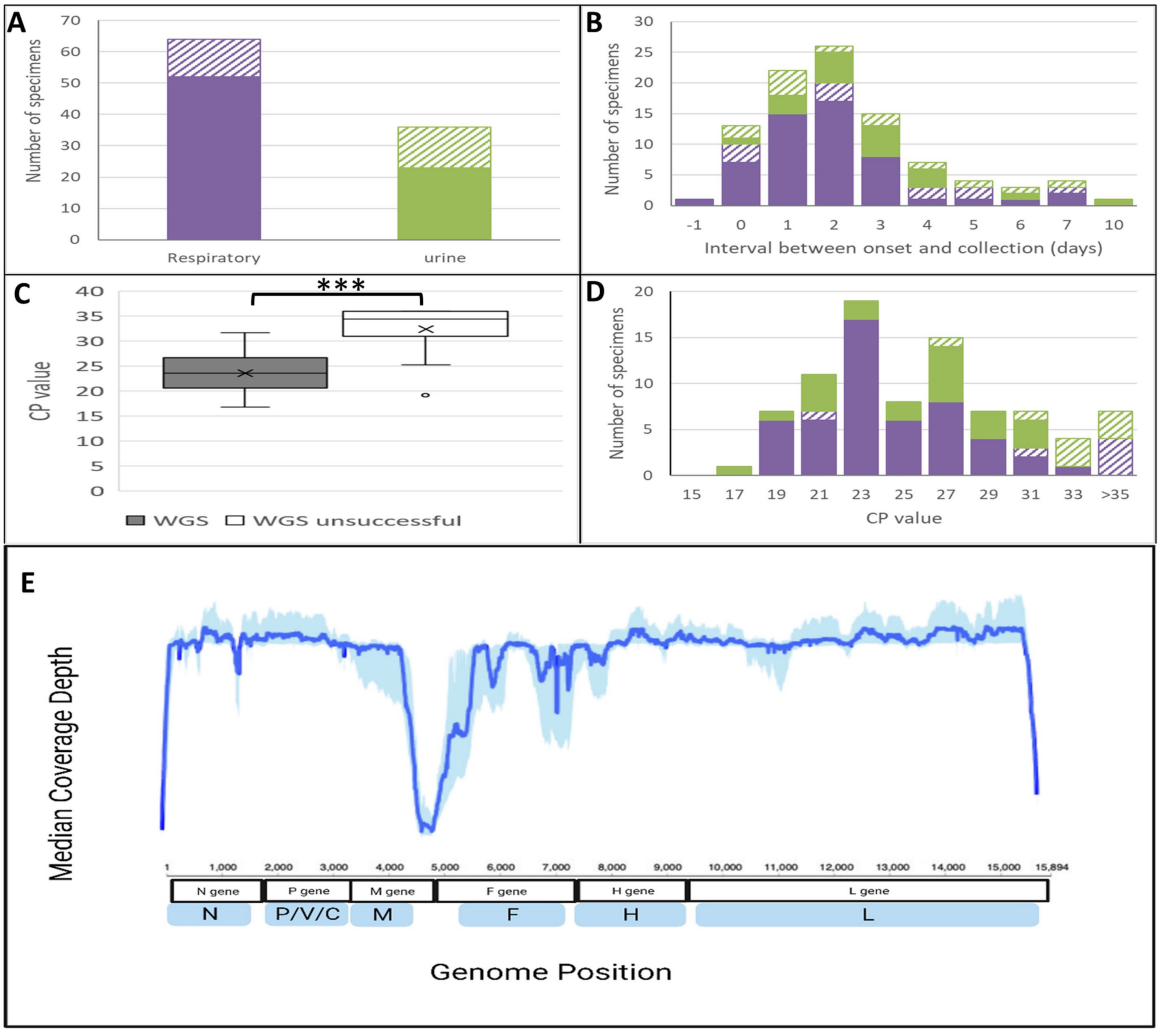
Characteristics of specimens and success of whole genome sequencing. (A–D) Filled bars indicate whole genome sequences achieved, while open or diagonal bars indicate whole genome sequencing failure. Purple color: respiratory specimens; green color: urine. (C) The asterisks indicate a significant difference between mean crossing point (CP) values (*t*-test) with a *p* value <0.0001. (E) Median coverage depth aggregated across 59 measles genomes (WGS-t) generated solely by Illumina sequencing. Depth coverage at each position was obtained through Galaxy using the tool SamTools depth for 59 genomes (samples with termini filled in and sanger patches were excluded from this). Sample termini were trimmed and proportions at each position were determined. Median (solid line) and 25th to 75th percentile (shaded area) values at each nucleotide position were plotted using ggPlot2 in R.

Whole genome sequencing using enrichment with MeV-specific probes and next-generation sequencing on an Illumina MiSeq instrument (Schulz et al., 2022) was attempted directly from clinical specimens for all cases from which an N450 sequence was obtained (*n* = 101). Whole genome sequences (minus the termini, termed WGS-t), with a depth of coverage of at least 10 reads across the entire 15,678 nucleotides, were obtained solely from the Illumina data for 59 specimens (58.4%). In general, a drop out was noted in the non-coding region between the matrix and fusion open reading frames (MF-NCR), which could be partially explained by the lack of RNA transcripts in this region (Figure 1E). This is also a difficult sequencing region due to the presence of stretches of homopolymers and secondary structure. For an additional fifteen specimens, the minimum sequencing depth of 10 was not achieved in the MF-NCR and was patched by the MF-NCR sequence obtained by conventional Sanger sequencing. For eight of these 15 patched specimens, there was less than 10-fold coverage in the first 10 nucleotides of the WGS-t (the start of the nucleoprotein ORF), a conserved region of the genome, which was also the case for an additional three specimens. In total, 77 (76.2%) MeV WGS-t sequences were obtained through a combination of Illumina sequencing and Sanger patching as needed (A line listing of the sequences, the composition of the WGS-t sequences, and accession numbers is provided in Supplementary Table S1).

Clinical specimens included respiratory (throat swabs or nasopharyngeal swabs) and/or urine samples (Figure 1A). There was a better rate of success for the respiratory specimens (81.3, 63.9% for the urine) which was not explained by the number of days between rash onset and specimen collection (Figure 1B). As expected, the CP/Ct value was inversely associated with the likelihood of obtaining complete WGS-t sequences: most successful specimens had a CP/Ct value <27 (third quartile value of 26.65) and unsuccessful specimens had CP/Ct values >30 (first quartile of 31.01) with a statistically significant difference between the means (*p* < 0.0001) (Figures 1C,D).

To determine the accuracy, the WGS-t sequences were compared to the sequences generated by Sanger methodology. In the N450 region, three out of 77 (3.9%) samples had a single nucleotide mismatch for an error rate of 8.7×10^−5^ per nucleotide, which would result in an expected 1.4 nucleotide differences per WGS-t. Fifty-nine (59) samples had both an Illumina WGS-t and a full-length Sanger MF-NCR (1,018 nucleotides in length). In this region, one sample (1.7%) had a single nucleotide mismatch (error rate of 1.6×10^−5^ per nucleotide), extrapolated to 0.3 nucleotide changes per WGS-t. Therefore, 0–1 errors in the nucleotide assignment could be expected in each MeV WGS-t obtained with this methodology.

The 21 genotype B3 and 56 genotype D8 WGS-t sequences were aligned by genotype and analyzed to identify hypervariable regions within the genome (Supplementary Figure S1). For both genotypes, the non-coding region between the matrix and fusion genes contained the maximum variability, seen also in other studies (Penedos et al., 2015; Vaidya et al., 2020; Song et al., 2022; Penedos et al., 2022; Cui et al., 2022; Beaty and Lee, 2016), followed by the 3′ end of the nucleoprotein open reading frame.

Sequence peculiarities were detected in three specimens. One genotype D8 specimen, MVs/Ontario.CAN/10.18 (GenBank accession OQ096298), had a unique single nucleotide insertion and deletion in the MF-NCR region. Two identical genotype B3 WGS-t sequences, the earlier one obtained from a measles case with recent travel history to the Philippines (MVs/British Columbia.CAN/4.19, GenBank OQ096316), had a cytosine residue at position 3,221 instead of an uracil which resulted in the loss of the stop codon of the phosphoprotein coding sequence. This novel sequence is described in a separate publication (Zubach et al., 2024).

### Description of cases, outbreaks, and sampling coverage

In total, 143 cases of measles were confirmed in Canada from 2018 to 2020 inclusive (Coulby et al., 2020; Coulby et al., 2021). The ages of the cases ranged from less than one year to 73 years, with a median age of 18. Most of the cases (70%) had not received any documented dose of measles-containing vaccine. Many cases were classified as imported (*n* = 59). The WHO global regions of travel, in descending order, were Western Pacific (*n* = 26, Philippines, Vietnam, multiple countries, and Cambodia), Europe (*n* = 11, Ukraine, multiple countries, France, Poland, Romania, and the United Kingdom), South East Asia (*n* = 11, India, Bangladesh, unspecified), the Americas (*n* = 5, USA, Brazil, and Uruguay), multiple regions (*n* = 3), Eastern Mediterranean (*n* = 2, Pakistan), and Africa (*n* = 1, Uganda) [Additional epidemiological details about the cases and outbreaks are provided in Coulby et al. (2020) and Coulby et al. (2021)].

**Figure 2:**
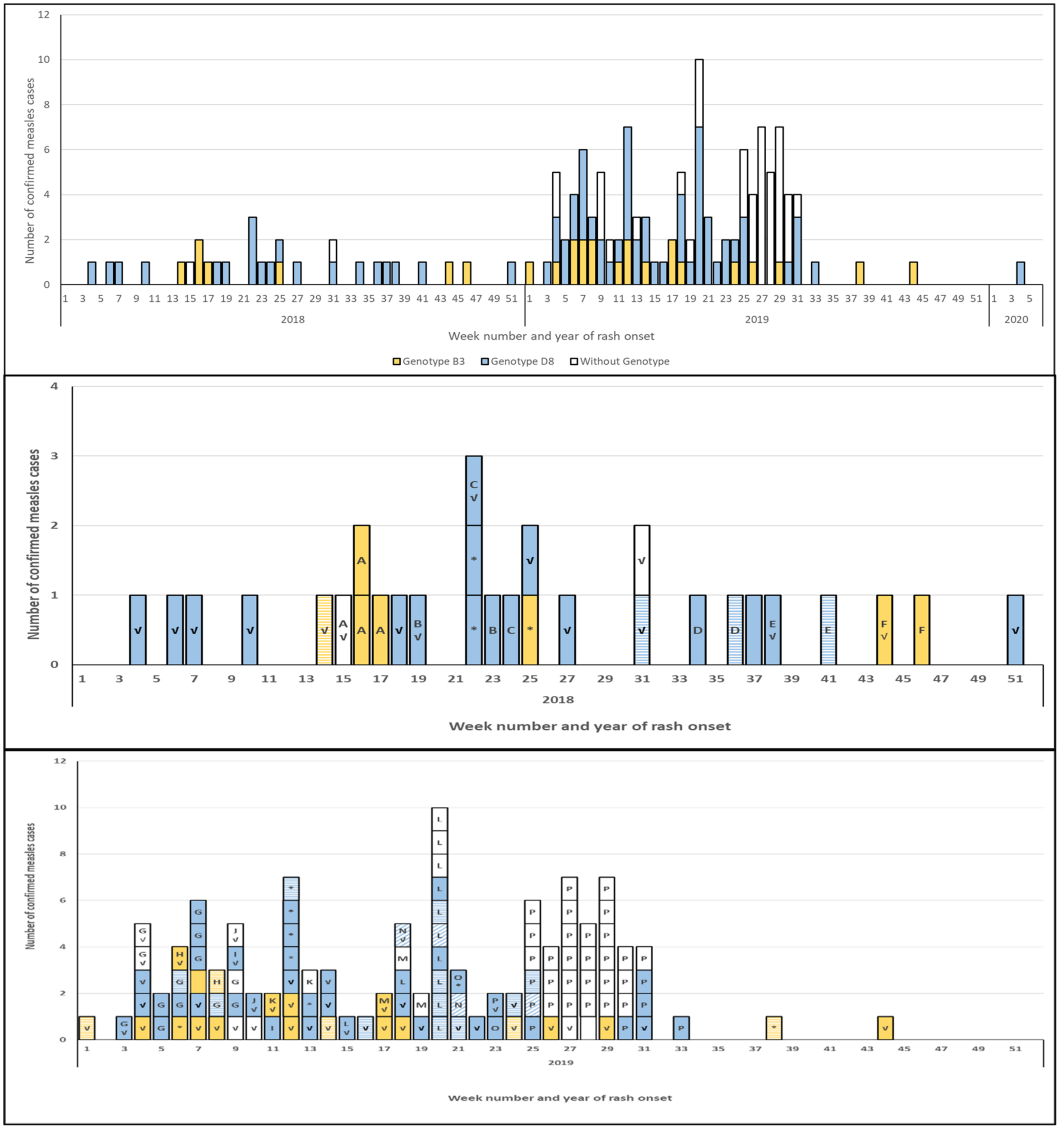
Epidemiological curve of confirmed measles cases, 2018–2020, by genotype and sequencing coverage. Top panel: all confirmed cases (*n* = 143). Middle panel: cases occurring in 2018 (*n* = 29). Bottom panel: cases occurring in 2019 (*n* = 113). All panels: blue-fill: genotype D8; yellow-fill: genotype B3; no fill: case without sequences. Bottom two panels: solid-fill: N-450, MF-NCR and WGS-t sequences obtained; diagonal pattern: N-450 and MF-NCR sequences obtained; horizontal pattern: N-450 sequence only. Letter indicates outbreak identifier; lack of a letter indicates isolated case. Check mark (√): case was imported. Asterisk (*): case with unknown source.

Most cases (*n* = 88) were associated with an outbreak (Table 1; Supplementary Figure S2). All outbreaks had at least one case for which the MeV genotype was determined, while 50 of 55 isolated cases (not associated with an outbreak) were genotyped, resulting in a genotype coverage of distinct chains of transmission of 93.0% (66/71). The size of the outbreaks ranged from two to 34 cases and most outbreaks (*n* = 13) had under five cases (average and median of 5.5 and two cases per outbreak respectively). An average of 84% of the cases in the outbreaks had a genotype determined. The largest and longest lasting outbreak, P, with 34 cases over 10 weeks, had the least number of viral sequences obtained (Table 1). However, both the start and the end of the outbreak were sampled (Figure 2).

**Table 1:** Summary of epidemiological information (shaded) and MeV sequence analysis of measles cases detected in Canada, 2018–2020.

All confirmed cases						N450 sequences			MF-NCR sequences		WGS-t sequences		
Outbreak	Earliest date	No. of cases	No. of weeks	Source (no. of cases)	Genotype (no. of cases)	DSId	No.	nt differences, min – max*	No.	nt differences, min – max*	No.	nt differences, min – max*	Summary of molecular epidemiology analysis
None	-	55	-	Imported (43), Unknown (12)	B3 (18), D8 (32)	multiple	18 32	0–13 0–21	13 27	0–45 0–55	13 27	0–265 0–315	Refer to Supplementary Figures
A	2018-04-11	4	2	Imported (3)	B3 (3)	4299	3	0–0	3	0–0	3	0–0	Phylogenetically confirmed distinct by N450
B	2018-05-12	2	4	Imported (1)	D8 (2)	5303	2	0	2	0	2	0	Phylogenetically confirmed distinct by N450
C	2018-05-31	2	2	Imported (1)	D8 (2)	4683	2	0	2	2	2	0	Cannot be phylogenetically distinguished from other similar strains; however, tMRCA excludes ongoing transmission from this earliest case in the MVs/Gir Somnath.IND/42.16 N450 clade
D	2018–08–21	2	2	Import related	D8 (2)	4221	2	0	1	-	1	-	Phylogenetically confirmed distinct by MF-NCR; tMRCA (WGS-t BEAST analysis) excludes direct link to phylogenetically similar outbreak E
E	2018-09-21	2	3	Imported (1)	D8 (2)	4742	2	0	1	-	1	-	Phylogenetically confirmed distinct by MF-NCR; tMRCA (WGS-t BEAST analysis) excludes direct link to phylogenetically similar outbreak D
F	2018–11-03	2	2	Imported (1)	B3 (2)	5683	2	0	2	0	2	0	Phylogenetically confirmed distinct by MF-NCR
G	2019-01-21	13	6	Imported (3)	D8 (10)	4683	10	0–0	8	0–1	8	0–5	Phylogenetically confirmed distinct by WGS-t; however, an outlier identified which likely does not belong to the outbreak
H	2019-02-17	2	2	Imported (1)	B3 (2)	5306	2	0	1	-	1	-	tMRCA (WGS-t BEAST analysis) excludes direct link to any other phylogenetically similar case
I	2019-03-04	2	2	Imported (1)	D8 (2)	5551	2	0	2	0	2	0	Phylogenetically confirmed distinct by WGS-t
J	2019-03-06	2	1	Imported (2)	D8 (1)	4683	1	-	1	-	1	-	WGS-t analysis provides evidence of larger outbreak with inclusion of additional contemporary cases of unknown source
K	2019–03–22	2	2	Imported (1)	B3 (1)	5622	1	-	1	-	1	-	N450 sequence distinct; tMRCA (WGS-t) excludes direct link to any other phylogenetically similar case
L	2019-04-21	12	5	Imported (1)	D8 (9)	4683	9	0–0	5	0–1	4	0–1	Phylogenetically confirmed distinct by WGS-t
M	2019-04-30	3	2	Imported (1)	B3 (1)	5852	1	-	1	-	1	-	N450 sequence distinct; tMRCA (WGS-t) excludes direct link to any other phylogenetically similar case
N	2019-05-12	2	3	Imported (1)	D8 (2)	4683	2	0	2	0	0	-	Cannot be distinguished by MF-NCR sequence; WGS-t could not be obtained
O	2019-06-01	2	2	Unknown	D8 (2)	4683	2	0	2	0	2	0	WGS-t analysis provides evidence of larger outbreak with inclusion of plausible index case
P	2019-06-13	34	10	Imported (1)	D8 (8)	4683	8	0–0	7	0–3	6	0–6	Phylogenetically confirmed distinct by WGS-t; however, an outlier identified which likely does not belong to the local outbreak.

^*^Minimum and maximum number of nucleotide differences between pairs of sequences.

### Phylogenetic analysis: use of MF-NCR sequences improves resolution over N450 sequences but is insufficient for frequently imported lineages

**Figure 3:**
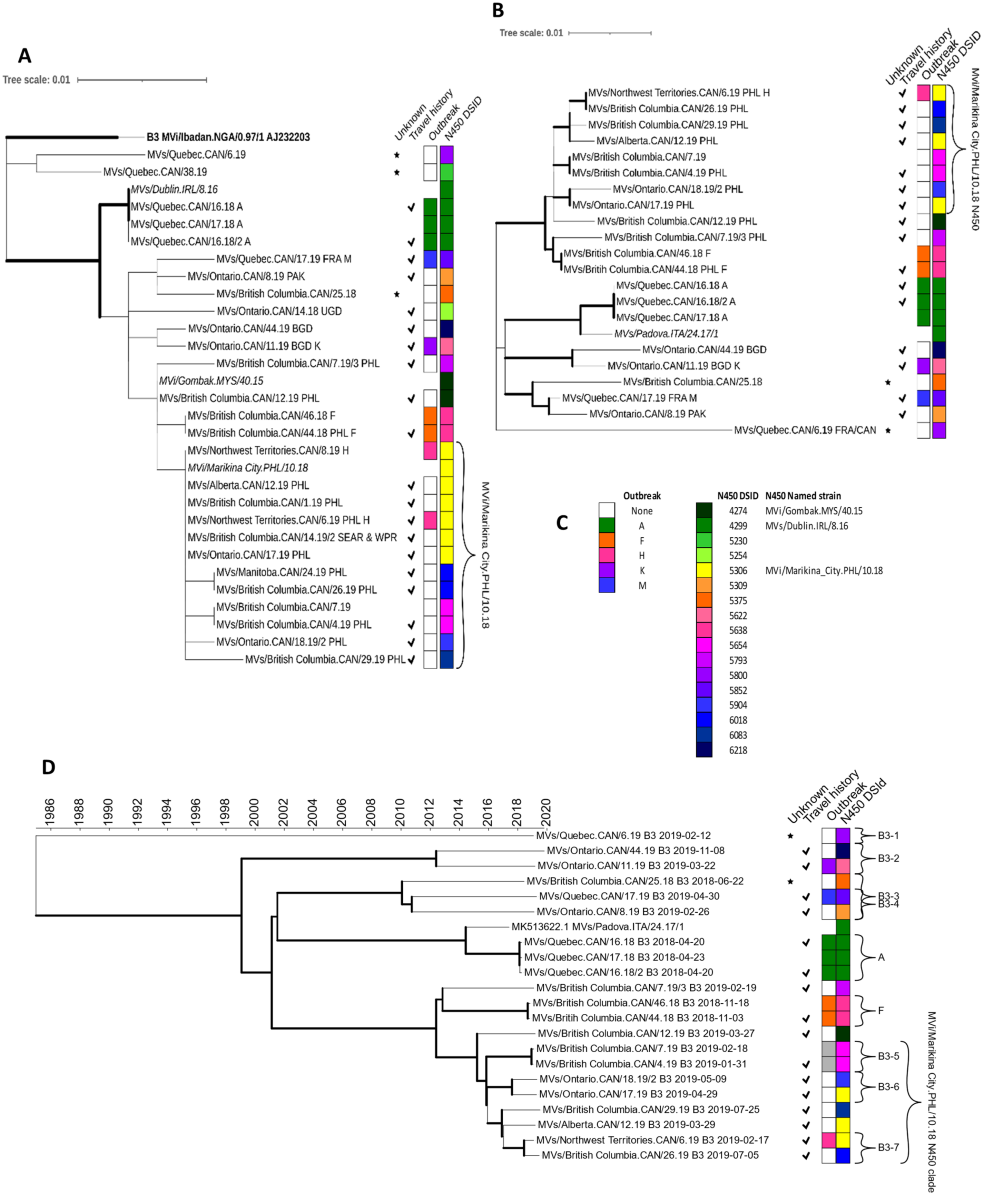
Phylogenetic analysis of measles genotype B3 strains detected in Canada, 2018–2020. (A,B) Maximum likelihood phylogenetic trees of the N450, 450 nucleotides, [(A) 27 sequences] and MF-NCR, 1018 nucleotides, [(B) 21 sequences] loci with 1,000 bootstrap replicates. Bold face indicates the genotype B3 reference sequence [N450 only (A)]. Italics are international sequences obtained from GenBank and for the N450 tree (A) represent WHO-named strains. Thick branch edges correspond to bootstrap values ≥0.7. (D) Maximum clade credibility phylogenetic tree of WGS-t sequences (*n* = 21) inferred with BEAST2. The time scale, in years, is at the top. Thick branch edges correspond to posterior values ≥0.7. Brackets with letters correspond to outbreaks and/or phylogenetic clusters analyzed in depth. Meta-data is provided in four columns to the right of the trees. A star symbol in the first column indicates a sequence from a case without a source identified. A check mark in the second column indicates that the case was considered imported. The two color strips indicate association with an outbreak and the MeaNS distinct sequence ID (DSId) assigned to the N450 sequence (including named strain, where applicable) with the legend provided in panel (C). The bracket present in all trees indicates the clade including the MVi/Marikina City.PHL/10.18 N450 named strain. (D) Shades of grey are added to the outbreak strip to denote the identification of related cases as a result of the sequence analysis. High resolution, interactive versions are available at: https://itol.embl.de/shared/GzTsavRiNVS9.

Of the 27 cases with genotype B3 MeV N450 sequences identified, most were single cases not associated with outbreaks (*n* = 18). The remaining nine cases were associated with five outbreaks for a total of 23 distinct chains of transmission (Supplementary Figures S2, S4). Many of the distinct epidemiological chains (11 of 23) were resolved by the analysis of the MeV N450 sequences into well-supported branches on the phylogenetic tree or into distinct sequences (Figure 3A; Supplementary Figure S3). Those that were not were contained in a phylogenetic clade originating with the MVi/Gomback.MYS/40.15 named strain and included as a major sub-clade, sequences highly similar (within 2 nucleotides) to the named strain MVi/Marikina City.PHL/10.18 (DSId 5306) (Figure 3A). The cases in this clade were primarily imported from the Philippines and occurred in 2019. The MVi/Marikina City.PHL/10.18 named strain was also reported from the US state of California in 2019, where it was associated with imports from the Philippines (Probert et al., 2021).

MF-NCR sequences (1,018 nucleotides) were obtained from 17 of the 23 chains of transmission, with much improved phylogenetic resolution (Figure 3B). Nine chains of transmission had been resolved by the N450 sequence and were confirmed by the MF-NCR phylogeny (outbreaks A, K, and M and six isolated cases) (Supplementary Figure S4). MF-NCR sequences were obtained from nine of the chains of transmission that could not be resolved by the N450 sequence and included six single, isolated cases and cases associated with two outbreaks (F and H) (Supplementary Figures S2, S3). MF-NCR sequences from outbreak F and three of the isolated cases (specifically with N450 DSIds 5306 and 5904), which were in the MVi/Marikina City.PHL/10.18 N450 phylogenetic clade, were phylogenetically resolved with good bootstrap support (>80%).

**Figure 4:**
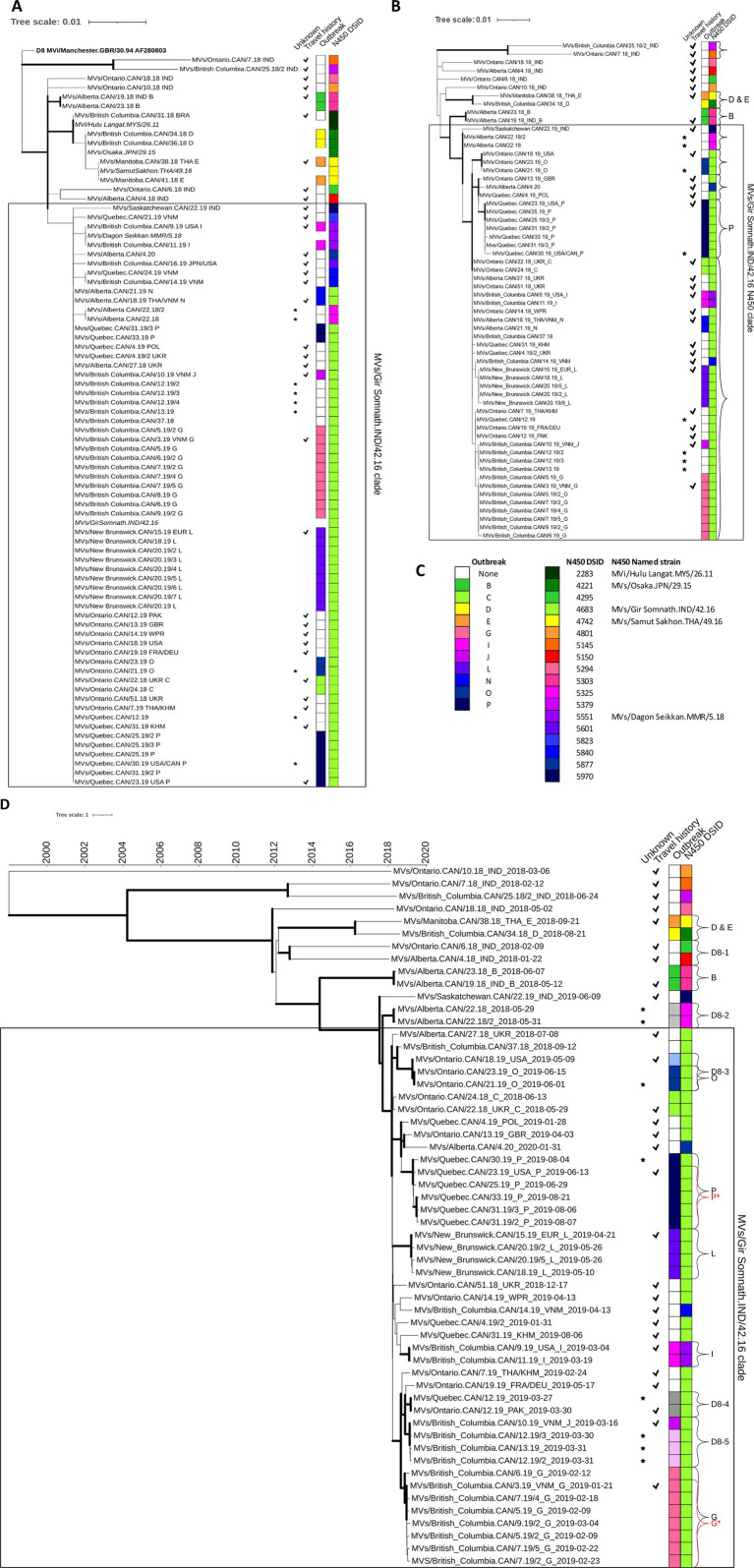
Phylogenetic analysis of measles genotype D8 strains detected in Canada, 2018–2020. (A,B) Maximum likelihood phylogenetic trees of the N450, 450 nucleotides, [(A) 74 sequences] and MF-NCR, 1018 nucleotides, [(B) 60 sequences] loci with 1,000 bootstrap replicates. Thick branch edges correspond to bootstrap values ≥0.7. (B) Curved brackets are clades with bootstrap values >70%. (D) Maximum clade credibility phylogenetic tree of WGS-t sequences (*n* = 56) inferred with BEAST2. The time scale, in years, is at the top. Thick branch edges correspond to posterior values ≥0.7. (B,D) Brackets with letters correspond to outbreaks and/or phylogenetic clusters analyzed in depth. (A,B,D) Annotations are as described for Figure 3 with the legend provided in panel (C). The box captures the sequences in the MVs/Gir Somnath.IND/42.16 N450 clade (D). Shades of grey and lighter-shaded colors are added to the outbreak strip to denote the identification of related cases as a result of the sequence analysis. High resolution, interactive versions are available at: https://itol.embl.de/shared/XLoW3xTqyafj.

In total, 43 epidemiological transmission chains, consisting of 32 single isolated measles cases and 11 outbreaks, ranging in size from two to 34 cases, had MeV genotype D8 sequences (total of 74 N450 sequences) (Table 1; Supplementary Figure S5). However, only 18 unique sequences (DSIds) were identified, which included five recognized as named strains (Figure 4A). The MVs/Gir Somnath.IND/42.16 named strain, which will be referred to as D8-Gir Somnath, was the only strain detected in both outbreak cases and single isolated cases (*n* = 17).

Phylogenetically, the bulk of the sequences (*n* = 61) and epidemiological chains (*n* = 33) were clustered in a clade that stemmed from and included D8-Gir Somnath (Figure 4A). The sequences in this clade were mostly identical and, with the exception of one imported sequence, which differed by 3–4 nucleotides (MVs/Saskatchewan.CAN/22.19), could not be resolved. The D8-Gir Somnath strain was also frequently detected globally beginning in 2018 and accounted for one-third of the submissions to the MeaNS database from 69 countries (Williams et al., 2022). Overall, only nine of the 43 transmission chains could be resolved by the N450 sequence: outbreak B and eight isolated cases (Supplementary Figures S2, S3).

The MF-NCR sequence (1,018 nucleotides) was obtained from 60 cases of measles genotype D8: 27 from isolated cases and the remainder associated with the 11 genotype D8 outbreaks for a total of 38 transmission chains. Overall, there was more diversity in the sequences and better support for the phylogenetic clades (Figure 4B). In total, only four outbreaks (B, D, E, and P) and six isolated cases were resolved (bootstrap value >70%) by the MF-NCR sequences.

For the 61 cases with N450 sequences in the MVs/Gir Somnath.IND/42.16 clade, 50 MF-NCR sequences were obtained. These sequences clustered together in a large clade with good support (bootstrap value of 88.1%) with improved but still limited diversity. When using a bootstrap cut-off of >70% for nodes, these 50 MF-NCR sequences obtained from 29 epidemiological chains could be resolved into only five distinct clusters (denoted by brackets in the MVs/Gir Somnath.IND/42.16 N450 clade in Figure 4B). However, only one phylogenetic cluster, with a bootstrap value of 86.3%, consisted of a single epidemiological chain: sequences from outbreak P (N450 DSId 4683). Overall, the MF-NCR sequence analysis was unable to provide much improvement to the resolution of distinct chains of transmission where the MVs/Gir Somnath.IND/42.16 N450 named strain was detected. This is in contrast to a study of outbreaks of D8-Gir Somnath in Spain occurring from 2019–2020 where the analysis of the MF-NCR sequence provided sufficient resolution to allow the identification of two simultaneous outbreaks not identified by the epidemiological investigation (Jacqueline et al., 2023).

### BEAST analysis of WGS-t corroborates epidemiological information from both linked cases and those without links

The 77 measles WGS-t were subjected to Bayesian evolutionary analysis by sampling trees (BEAST), which incorporates time (Supplementary Table S2). All branches in the genotype B3 maximum clade credibility (MCC) tree had strong posterior support (≥73%) and all five outbreaks were phylogenetically resolved, including outbreak H which had not been resolved with the MF-NCR sequence (Figure 3D; Supplementary Figure S2). The genotype D8 analysis also had strong posterior support for many of the branches (Figure 4D). A detailed analysis of the epidemiological and phylogenetic clusters was performed to see if the BEAST dating was consistent with the information obtained by the epidemiological investigation (Table 2). For the two genotype B3 outbreaks (A and F) with at least two WGS-t sequences (Table 1), the inferred emergence of the most recent common ancestors (tMRCA) encompassed the onset date of the documented index cases, consistent with onward transmission to the secondary case(s).

**Table 2:** Estimated dates of most recent common ancestors (tMRCA) inferred by BEAST for clusters of measles cases of interest, grouped by epidemiologically defined status.

Genotype	N450 DSID	Description (WGS-t phylogenetic tree label)	Onset date of index^a^ case	No. of WGS-t sequences in the clade	nt differences, min – max*	Earliest onset date in the clade	tMRCA (95% HPD)	Comments
Epidemiologically defined clusters: intra-outbreak								
B3	4299	Outbreak A (A)	2018.2767	3	0–0	2018.3014	2018.1485 (2017.9593–2018.3010)	tMRCA consistent with transmission within the clade
	5638	Outbreak F (F)	2018.8411	2	0	2018.8411	2018.7141 (2018.5277–2018.8411)	tMRCA consistent with transmission within the clade
D8	5303	Outbreak B (B)	2018.3616	2	0	2018.3616	2018.3132 (2018.2154–2018.3616)	tMRCA consistent with transmission within the clade
	4683	Outbreak G (G)	2019.0575	8	0–5	2019.0575	2018.8909 (2018.7522–2019.0146)	tMRCA puts index case 16 (≈one generation) – 111 days earlier
	4683	Outbreak G subclade that excludes MVs/British Columbia.CAN/6.19 (G*)	2019.0575	7	0–1	2019.0575	2018.9848 (2018.900–2019.0574)	Given the gap of 0.5 day, tMRCA likely does not exclude transmission within the subclade. Infers MVs/British Columbia.CAN/6.19 is an outlier (is not part of the outbreak).
	5551	Outbreak I (I)	2019.1726	2	0	2019.1726	2019.1233 (2019.0250–2019.1726)	tMRCA consistent with transmission within the clade
	4683	Outbreak L (L)	2019.3041	4	0–1	2019.3041	2019.2313 (2019.1286–2019.3041)	tMRCA consistent with transmission within the clade
	4683	Outbreak O (O)	2019.4164	2	0	2019.4164	2019.3853 (2019.3322–2019.4164)	tMRCA consistent with transmission within the clade
	4683	Outbreak P (P)	2019.4493	6	0–6	2019.4493	2019.3233 (2019.1849–2019.4436)	tMRCA puts index case two – 97 days earlier
	4683	Outbreak P but excluding MVs/Quebec.CAN/30.19 (P*)	2019.4493	5	0–4	2019.4493	2019.3477 (2019.2167–2019.4493)	tMRCA consistent with transmission & infers MVs/Quebec.CAN/30.19 is an outlier (is not part of the local outbreak)
Epidemiologically defined clusters: between outbreak and unrelated cases								
B3	5622 & 6218	Phylogenetic node containing outbreak K and epidemiologically distinct, imported case (B3-2)		2	54	2019.2219	2012.4406 (2007.1502–2017.1502)	tMRCA inconsistent with transmission within the clade
	5309 & 5852	Phylogenetic node containing outbreak M and epidemiologically distinct, imported case (B3-4)		2	62	2019.15616	2010.8075 (2005.0161–2017.0772)	tMRCA inconsistent with transmission within the clade
	5,306 & related	Outbreak H sequence and sequences from seven non-outbreak cases (MVi/Marikina City.PHL/10.18 N450 clade)		8	0–32	2019.0849	2015.8777 (2013.3407–2018.4831)	tMRCA inconsistent with transmission within the clade
	5306 & 5904	Two contemporary imported cases in the same province (B3-6)		2	13	2019.3260	2017.6390 (2016.2377–2019.0281)	tMRCA inconsistent with transmission within the clade
	5306 & 6018	Phylogenetic node containing outbreak H and epidemiologically distinct, imported case (B3-7)		2	5	2019.1315	2018.4630 (2017.8130–2019.1059)	tMRCA inconsistent with transmission within the clade
D8	4221 & 4742	Outbreaks D and E (D & E)		2	50	2018.60274	2016.2707 (2015.3547–2017.1221)	tMRCA inconsistent with transmission within the clade
	4295 & 5150	Two cases imported from the same country (D8-1)		2	113	2018.06027	2012.8272 (2011.0598–2014.4269)	tMRCA inconsistent with transmission within the clade
	4683 & related	Phylogenetic clade containing outbreaks C, G, J, L, N, O, and P sequences and sequences from 21 non-outbreak cases (MVs/Gir Somnath.IND/42.16 clade)		43	0–37	2018.4137	2018.2115 (2018.0057–2018.3852)	tMRCA inconsistent with transmission within the clade
Investigation of unknown source cases								
B3	5800	Isolated case of unknown source (B3-1)		22	0–274	2018.30137	1988.8057 (1967.1927–2010.8295)	No phylogenetically similar sequences; the tMRCA is for the entire B3 WGS-t tree; excludes link to any other case
	5375	Phylogenetic node containing isolated case of unknown source and two other distinct, imported cases (B3-3)		3	62–73	2018.47397	2010.0984 (2003.5064–2016.8090)	Phylogenetically distinct; tMRCA inconsistent with transmission
	5654	Two sequential cases in the same province with the later case of unknown source (B3-5)	2019.08493	2	0	2019.0849	2018.9732 (2018.8068–2019.0849)	tMRCA consistent with transmission within the clade
D8	5325	Two contemporary cases of unknown source in the same province (D8-2)	2018.3699	2	1	2018.40822	2018.3083 (2018.1700–2018.4055)	tMRCA consistent with unknown common ancestor of both cases
	4683	Two epi-related cases, outbreak O, of unknown source and one earlier imported case, putative index, in same province (D8-3)	2019.3534	3	0–1	2019.3534	2019.2943 (2019.1968–2019.3534)	tMRCA consistent with transmission within the clade
	4683	Phylogenetic node containing two contemporary cases, one of unknown source (D8-4)	2019.19726	2	0	2019.23562	2019.2043 (2019.1426–2019.2356)	tMRCA consistent with unknown common ancestor of both cases
	4683	Phylogenetic node containing outbreak J sequence and three later cases in the same province of unknown source. Outbreak J index case not sequenced (D8-5)	2019.1781	4	0	2019.2055	2019.15 (2019.0722–2019.2055)	tMRCA consistent with transmission within the clade

^aThe onset date of the known index case for epidemiologically confirmed outbreaks. For phylogenetic clusters of cases not known to be related, the date is for a putative index case, either estimated based on an average incubation time of 14 days if no suspect index case or of a confirmed case suspected to be the index case.^

For genotype D8 outbreaks, six had at least two WGS-t sequences and enough phylogenetic information to perform BEAST analysis: B, G, I, L, O, and P. All but outbreak P were well characterized epidemiologically and genetically. Four belonged phylogenetically to the MVs/Gir Somnath.IND/42.16 N450 clade (Figure 4D). For outbreaks B, I, L, and O, the 95% HPD interval of the tMRCAs encompassed the onset dates of the known index cases for these outbreaks, consistent with onward transmission from the index case to the other outbreak cases that were sequenced (Table 2).

In addition to applying BEAST to epidemiologically related cases, we tested the reliability of the analysis to exclude relatedness of cases with phylogenetically similar WGS-t but which were epidemiologically distinct (Table 2). Five small outbreaks (D, E, H, K, and M) each had only one WGS-t and each phylogenetically clustered with one other sequence that was from an epidemiologically distinct case (Figures 3D, 4D). In each case, the estimated date of the common ancestor for the pairs of sequences predated the onset date of earliest case of the pair by at least half a year, allowing for the confident exclusion of undetected links between the pairs of cases (Table 2).

Both genotypes B3 and D8 had many sequences from epidemiologically distinct cases that had highly similar N450 sequences: the genotype B3 MVi/Marikina City.PHL/10.18 N450 clade and the D8 MVs/Gir Somnath.IND/42.16 N450 clade (Figures 3D, 4D). For the genotype B3 MVi/Marikina City.PHL/10.18 N450 clade, which included WGS-t sequences from seven distinct, imported cases, the internal node age of the most recent common ancestor (tMRCA) was 2015.8777, which long predated the importation of these cases detected in Canada (earliest onset date of 2019.0849). For the genotype D8 MVs/Gir Somnath.IND/42.16 N450 clade, which consisted of sequences from seven outbreaks and 21 isolated cases, the tMRCA excluded the onset date (fractional year date of 2018.4137) of the earliest detected case, an importation from Ukraine, for this clade and thus was inconsistent with continuing onward transmission of this measles strain within Canada from this initial introduction.

The D8-Gir Somnath lineage, which was repeatedly imported into Canada between 2018 and 2020, was also being detected globally. To explore the evolution of this lineage and the relationship of Canadian sequences to those detected elsewhere, we obtained 25 measles genotype D8 WGS-t sequences with N450 sequences identical to DSID 4683 and with WHO strain names from GenBank (providing location and date information). These sequences were detected in cases in China and the USA in 2019 (Zhang et al., 2020; Probert et al., 2021). MCC phylogenetic analysis placed the international sequences in the clade with the Canadian DSID 4683 sequences without changing the node for the MRCA. The international sequences were scattered throughout the clade and clusters within this clade were composed of both Canadian and international sequences, as opposed to a distinct geographic clustering pattern, further validating the conclusion that the D8-Gir Somnath sequences detected in Canada were due to repeated importations rather than local circulation (Supplementary Figure S7).

### Application of genomic analysis to a large under-sampled D8 – Gir Somnath measles outbreak with missing epidemiological information

Outbreak P was the largest outbreak and included many cases for which specimens were not available (Table 1). The outbreak included community spread and had ties to a large, long-lasting outbreak in the USA (Coulby et al., 2021; Zucker et al., 2020). At least one case within the affected community could not have the source of their infection conclusively attributed as they had connections within the outbreak and had recently traveled to the related community in the USA. As a result, there were gaps in the epidemiological understanding of the outbreak such as the number of generations (Coulby et al., 2021). In addition, many of the cases were identified retrospectively by serology, beyond the window for conducting viral sampling, leading to an incomplete molecular epidemiological picture (N450 sequences from only eight of 34 cases) (Figure 2). Even with this under-representation (six of 34 or 17.6% with WGS-t), the WGS-t phylogenetic analysis accurately and with high confidence (posterior value of 0.9998) placed the sequences from this outbreak separately and distinctly from sequences from epidemiologically unrelated cases (Figure 4D), even though the number of nucleotide differences was as low as 10 between unrelated cases (pairwise comparison between the index case MVs/Quebec.CAN/23.19 and the phylogenetically closest sequence, MVs/Quebec.CAN/4.19).

The BEAST analysis of the WGS-t associated with outbreak P estimated the tMRCA to be two to 97 days earlier than that of the earliest confirmed case that had a known travel history (MVs/Quebec.CAN/23.19), suggesting either a longer-than-expected incubation period of the index case, an earlier, undetected initiation of this outbreak, or the existence of more than one chain of transmission (Table 2) (This gap remains with the addition of the international but unrelated sequences). The outbreak included at least one case, MVs/Quebec.CAN/30.19, for which the source was unclear (imported or infected as part of the transmission chain from the first identified case). This case also had the maximum number of nucleotide differences (two–six) in the pairwise analysis of the outbreak WGS-t sequences, while the other pairs had between zero and four differences, suggesting that it was part of a separate chain of transmission. The MCC tree had a sub-clade that excluded MVs/Quebec.CAN/30.19 (Supplementary Figure S8, and P* in Figure 4D). The resulting sub-clade had a tMRCA 95% HPD interval that overlapped with the onset date of the earliest known case (MVs/Quebec.CAN/23.19), suggesting that the outlier was indeed the result of infection in the related outbreak in the USA (Table 2). The addition of sequences from the related outbreak in the USA would help to clarify the molecular epidemiology analysis but were not available.

For comparison, the only other sizeable outbreak in this study period, L, another D8-Gir Somnath lineage outbreak, had a single index case with very clear chains of transmission established that included three generations (Coulby et al., 2021) and no more than one nucleotide difference between pairs of sequences (Table 1). Here, the BEAST tMRCA was consistent with the onset date of the index case (Table 2).

### BEAST analysis improves resolution in outbreaks with multiple chains of transmission

Outbreak G had three co-index cases who had traveled to Vietnam together (Coulby et al., 2021) and had onset dates within nine days of each other (Supplementary Figure S8). There was a total of five generations attributed to the outbreak, which primarily occurred in two schools (Coulby et al., 2021). Only one of the three index cases, the earliest with respect to rash onset, had specimens available for sequencing: MVs/British Columbia.CAN/3.19 (abbreviated as BC/3.19). The tMRCA for the phylogenetic cluster containing outbreak G was estimated to be at least 16 days earlier than that of the rash onset for BC/3.19 (Table 2). Inspection of the MCC tree revealed a subclade that included BC/3.19 and all subsequent outbreak G sequences with the exception of one: MVs/British Columbia.CAN/6.19 (Figure 4D, G and G* and Supplementary Figure S8). This outlier sequence, abbreviated as BC/6.19, had the most nucleotide differences from sequences obtained from the other cases attributed to the outbreak (four–five nt differences). The sequences from the other cases were identical to BC/3.19, with the exception of the case representing the last generation in the outbreak (MVs/British Columbia/9.19/2), which differed by only a single nucleotide (noting also that one nucleotide could be due to the sequencing methodology). Thus, the BC/6.19 WGS-t is an outlier sequence with a number of nucleotide differences that cannot be accounted for by the passage of time. In addition, a tool developed by Penedos et al. for predicting the number of nucleotide changes over time using the Poisson distribution estimates the likelihood of observing four differences with seven cumulative weeks of evolution between BC/3.19 and BC/6.19 at 0.039 (Penedos et al., 2022). Furthermore, the branch point for BC/6.19 from the G* sub-clade precedes in time that of the sequenced index case, BC/3.19, with a posterior value of 1, which is inconsistent with onward transmission from BC/3.19 to BC/6.19 (Supplementary Figure S8). Therefore, it is unlikely that BC/6.19 was part of the same transmission chain as BC/3.19 and the other sequenced cases. Instead, the data suggests that BC/6.19 was the result of transmission from an unidentified case that shared a proximate common ancestor to BC/3.19, likely as another import event. There were 11 documented imported measles cases associated with travel to Vietnam in 2019, only six of which had measles N450 sequences obtained (Coulby et al., 2021). However, no other case or cases captured by the surveillance system, sequenced or not, were identified as possibly being connected to BC/6.19 either through geographic and/or temporal proximity or by sequence analysis, and so an alternative source cannot be identified.

### Investigation of cases of unknown source: BEAST analysis of WGS-t demonstrates links

Of the 143 measles cases included in this study, a source of infection (travel or connection to another case) could not be identified for 13 cases (Supplementary Figure S6). For one case, MVs/Quebec.CAN/38.19, only the N450 sequence (DSId 5230) could be obtained, which was distinct from all other genotype B3 sequences in the study (pairwise distances of six– 11 nucleotides) such that it was unlikely to be related to any other case of genotype B3 (Supplementary Figure S3). WGS-t sequences were available for the remaining 12 cases. Two genotype B3 sequences (MVs/Quebec.CAN/6.19, DSId 5800 and MVs/British Columbia.CAN/25.18, DSId 5375) were well resolved phylogenetically (“B3-1” and “B3-3” in Figure 3D). They had distinct N450 sequences (Supplementary Figure S3) and their BEAST-estimated node age for the MRCA was at least 7 years prior, excluding links to other cases (Table 2). A genotype D8 sequence, MVs/British Columbia.CAN/37.18, belonging to the D8-Gir Somnath N450 clade, had strong phylogenetic resolution with the WGS-t (posterior of 0.999) (Figure 4D).

The WGS-t sequences from the remaining nine cases of unknown source clustered phylogenetically with sequences from other contemporaneous cases, and links within these clusters were explored by BEAST. In three phylogenetic clusters, there were both sequences of unknown source and sequences from imported, or import-related, cases with earlier onsets (“B3-5” in Figure 3D and “D8-3” and “D8-5” in Figure 4D). Cluster B3-5 consisted of an imported case and an identical sequence from a case occurring three weeks later in the same province and were the only two cases with N450 DSId 5654. The onset date for the imported case was encompassed by the confidence interval for the BEAST estimated date for the MRCA, consistent with transmission from the imported case to the later case of unknown source (Table 2). These two WGS-t sequences also shared the novel loss of stop codon mutation in the phosphoprotein gene (Zubach et al., 2024).

The second cluster (“D8-3” in Figure 4D) included two sequential, epidemiologically linked cases of unknown source (outbreak O) and an earlier imported case (MVs/Ontario.CAN/18.19) as a putative source case for the outbreak O cases. These sequences belonged to the D8-Gir Somnath lineage (N450 DSId 4683). There was one nucleotide difference in the WGS-t sequence between the putative source case and the later two outbreak O cases, which had no differences between them. The onset date of the imported case was 2019.3534 and occurred 23 days earlier than the index case of outbreak O. The estimated tMRCA was 2019.2943 with a 95% HPD interval (2019.1968 to 2019.3534) that included the onset date of the putative source case, consistent with this case transmitting to the outbreak O index case (Table 2).

The third cluster (“D8-5” in Figure 4D) consisted of four cases that included the single outbreak J sequence (MVs/British Columbia.CAN/10.19, the secondary case of the outbreak) and three cases unknown source, each occurring 14 or 15 days after MVs/British Columbia.CAN/10.19. The epidemiological investigation had only identified two cases as belonging to the outbreak. However, WGS-t sequences were identical among all four of the sequenced cases. We postulated that these three cases of unknown source but with identical WGS-t were part of outbreak J through some unknown link. Thus, we used the outbreak J index case, which was not sequenced but had an onset of 2019.1781, as the index case for the analysis. The onset for the sequenced (secondary case) outbreak J case was 2019.2055. The estimated tMRCA was 2019.1500 with a 95% HPD interval (2019.0722 to 2019.2055) that included the onset date of both outbreak J cases, supporting the hypothesis.

The remaining 3 WGS-t sequences from cases of unknown source clustered into two phylogenetic clusters, “D8-2” and “D8-4” (Figure 4D). The D8-2 cluster consisted of two cases of unknown source that occurred in the same province and with onset in the same week. This cluster had identical N450 (DSId 5325) and MF-NCR sequences and deviated by only one nucleotide in the WGS-t. The phylogenetic analysis had not produced a putative index case (inclusive of all sequenced cases in all provinces), therefore a missing index case was inferred, and an onset date of 2018.3699 was estimated based on an average incubation time of 14 days (one generation earlier). The BEAST-inferred tMRCA was 2018.3083 with a 95% HPD interval (2018.1700 to 2018.4055) that included the estimated onset date, suggesting that both cases were exposed to the same unknown common source case that was not detected by the surveillance system. The detected cases occurred during the start of the summer tourism season and the unknown index case could have been a tourist who may have not presented to the surveillance system or may have left the country while still in the prodromal, yet infectious, stage.

The final unknown source case had a genotype D8 WGS-t sequence that was identical to that of a case recorded as imported occurring in the same week but detected in a neighboring province (“D8-4” in Figure 4D). While they both shared the D8-Gir Somnath N450 sequence (DSId 4683) imported multiple times in this time period, the finding of identical WGS-t sequences is unlikely to occur by chance. The 95% HPD interval of the BEAST tMRCA encompassed the onset date of a putative ancestor two weeks prior, strongly supporting the existence of a common source for the two cases (Table 2). A review of the information for both cases revealed that both had returned from international travel on the same day (2019.1890), which was also within the 95% HPD interval, revealing a possible common exposure location. Furthermore, one case had returned from a country with verified measles elimination status not known to be experiencing an outbreak (thus was recorded as unknown source) while the other had returned from a country where genotype B3 was the endemic virus. Although residing in neighboring provinces, given their regional locations, it is plausible that they arrived in Canada at the same major international airport. However, airport information was unavailable and so we were unable to explore this possibility further. The scenario of a hypothetical common airport exposure also emerged from the phylogenetic analysis of WGS-t of three D8-Gir Somnath cases in California in 2019 (Probert et al., 2021). The cases shared nearly identical WGS-t sequences and had traveled through the same airport on the same day, but BEAST analysis to interrogate the likelihood was not performed.

### Pairwise comparisons of MF-NCR and WGS-t sequences: number of nucleotide differences not always sufficient to distinguish between unrelated cases of the same lineage

Pairwise distances were calculated for genotype B3 and D8 MF-NCR and WGS-t sequences, stratified by related status, either epidemiologically or as determined by BEAST analysis of the WGS-t (Table 3; Supplementary Figure S9). Sequence pairs from cases that were known to be related, either directly or within the same outbreak, had very similar sequences. The MF-NCR was often identical, with a maximum of one nucleotide difference that was noted in the longest lasting outbreak. The WGS-t sequences from epidemiologically related pairs of cases had a mean of one nucleotide difference and a range of zero to four differences. Cases not known to be related were more likely to have more nucleotide differences between their sequences (Mann–Whitney test, *p* < 0.0001), but even pairs of cases confirmed to be not related could have very similar sequences, particularly if they were of the same N450 lineage. A study of genotype B3 WGS-t, primarily of the named strain MVi/MarikinaCity.PHL/10.18 as seen in our study, from cases detected in the US state of California in 2019 noted that a minimum of seven nucleotide differences between WGS-t sequences could distinguish cases as being epidemiologically not related (Probert et al., 2021). Another American study that analyzed pairs of measles (genotype B3) cases directly linked (two pairs) or not linked (five pairs) that occurred in evacuees from Afghanistan in 2021 found that pairs of linked cases had no more than two nucleotide differences across the genome, while pairs without links had as little as one nucleotide difference with a maximum of 105 (Masters et al., 2023). In our dataset, genotype B3 WGS-t sequences from related cases were identical (five pairs) while pairs of unrelated cases differed by at least five nucleotides, with an average of 120 (205 pairs).

**Table 3:** Summary of pairwise distances stratified by genotype and related status between cases (epidemiologically or WGS-t).

Genotype	Description	MF-NCR			WGS-t sequences		
		No. of pairs	Mean p-distance (min, max)	Mean no. nt differences (min, max)	No. of pairs	Mean p-distance (min, max)	Mean no. nt differences (min, max)
B3	All pairs	210	0.0195 (0, 0.0440)	20 (0, 45)	210	0.0075 (0, 0.0175)	117 (0, 274)
	Related pairs	5	0 (0, 0)	0 (0, 0)	5	0 (0, 0)	0 (0, 0)
	Unrelated pairs	205	0.0200 (0, 0.0440)	20 (0, 45)	205	0.0077 (0.0003, 0.0175)	120 (5, 274)
D8	All pairs	1770	0.0097 (0, 0.0538)	11 (0, 58)	1,540	0.0037 (0, 0.0201)	58 (0, 315)
	Related pairs	61	0.0001 (0, 0.0010)	0 (0, 1)	51	0 (0, 0.0003)	1 (0, 4)
	Unrelated pairs	1709	0.0107 (0, 0.0566)	11 (0, 58)	1,489	0.0038 (0.0001, 0.0201)	60 (2, 315)
	Unrelated pairs, Gir Somnath	845	0.0036 (0, 0.0090)	4 (0, 9)	693	0.0009 (0.0001, 0.0019)	14 (2, 29)
B3 & D8	All pairs	1980	0.0114 (0, 0.0566)	12 (0, 58)	1750	0.0041 (0, 0.0201)	65 (0, 315)
	Related pairs	66	0.0001 (0, 0.0010)	0* (0, 1)	56	0 (0, 0.0003)	1* (0, 4)
	Unrelated pairs	1914	0.0118 (0, 0.0566)	12* (0, 58)	1,694	0.0043 (0.0001, 0.0201)	67* (2, 315)
	Unrelated pairs with identical N450	848	0.0036 (0, 0.0090)	4 (0, 9)	696	0.0009 (0.0001, 0.0019)	14 (2, 29)

^*^Mann–Whitney test, *p* < 0.0001.

### Limitations

This study had some limitations. Notably, the under-sampling (17.6% of the reported cases had a WGS-t sequence obtained) and poorly defined epidemiological parameters of the largest outbreak constrained the ability to interpret the BEAST analysis. This might have been remedied by the addition of sequences from the connected outbreak in the USA, but they were not available. Nonetheless, the phylogenetic analysis of both the MF-NCR and WGS-t loci was sufficient to differentiate the cases associated with this outbreak from other cases also of the D8-Gir Somnath N450 lineage and to conclusively attribute the source of one case for which more than one source (import or local acquisition) was possible. The lack of sequences from all cases in other outbreaks, such as outbreak J, prevented us from confirming the inferences made when interpreting the outcome of the BEAST analysis.

## Conclusion

We were able to apply our MeV NGS sequencing approach to clinical specimens (throat/nasopharyngeal specimens and urine) with a success rate of 75% but a drop-out in sequencing coverage in the MF-NCR was observed, which sometimes required Sanger sequencing to overcome, and has also been noted in other studies (Penedos et al., 2015; Probert et al., 2021; Bucris et al., 2024; Masters et al., 2023). The likelihood of success did not appear to be correlated with specimen type and only loosely with the interval between rash onset and collection date. However, there appeared to be a correlation with the viral load, with specimens having a CP/Ct value below 30 more likely to generate full WGS-t sequences.

Phylogenetic analysis of loci beyond the N450 was able to provide additional genetic resolution to epidemiologically defined clusters. Most chains of transmission (40 of 59 with WGS-t sequences) were well resolved by the WGS-t phylogenetic analysis, with strong support (posterior values ≥0.7). The addition of geographically distinct yet phylogenetically similar sequences in the case of genotype D8 helped demonstrate the absence of locally circulating strains. The BEAST analysis further confirmed that the two most frequently detected lineages (phylogenetically similar to the B3 named strain MVi/Marikina City.PHL/10.18 and the D8 named strain MVs/Gir Somnath.IND/42.16) were the result of repeated importations.

Of the 16 outbreaks occurring during the study period, the molecular epidemiology analysis, sometimes already at the level of the N450 sequence, was able to conclusively corroborate the epidemiological information for 13, reproducing the utility and outcomes that have been demonstrated in many other studies (Bianchi et al., 2019; Cheng et al., 2023; Wang et al., 2023; Magurano et al., 2019; Seki et al., 2019; Augusto et al., 2019). This study built upon other studies that have used BEAST analysis at the level of large phylogenetic clades to explore evidence of local transmission and harnessed the node-dating capacity of BEAST to interrogate single transmission chains. In this way, the BEAST analysis of the WGS-t was able to convincingly demonstrate the expansion of two outbreaks by the inclusion of additional contemporary cases for which the epidemiological investigation had been unable to identify links. Furthermore, this analysis revealed, with high probability, the existence of three additional unrecognized outbreaks among the cases categorized as unknown source. However, it is not always possible to obtain sufficient genetic information from all cases; in this analysis, one outbreak (N) remains virologically unconfirmed due to a lack of data. Viral specimens are not always obtained or are not conducive to MeV sequencing for a variety of reasons. Furthermore, the processes for obtaining MF-NCR and WGS-t sequences are technically more challenging, add additional cost, and the analysis, particularly BEAST, requires sophisticated computing capability and bioinformatics expertise, making it unsuitable for all settings. Therefore, there continues to be a need for traditional epidemiological investigations of all cases, including in elimination setting, and for this information to be integrated with the laboratory-derived data.

## Data Availability

The datasets presented in this study can be found in online repositories. The names of the repository/repositories and accession number(s) can be found in the article/Supplementary material.

## Footnotes

^1^Available at: https://www.bioinformatics.babraham.ac.uk/projects/fastqc/.

^2^Available at: http://tree.bio.ed.ac.uk/software/figtree/.
