# Supplementary material for: Genomic tools for post-elimination measles molecular epidemiology using Canadian surveillance data from 2018–2020
Source: Front Microbiol. 2024 Nov 19;15:1475144. doi: 10.3389/fmicb.2024.1475144 (PMC11611582; doi:10.3389/fmicb.2024.1475144)
Supplement: Supplementary file 1 [file Presentation_1.PDF]

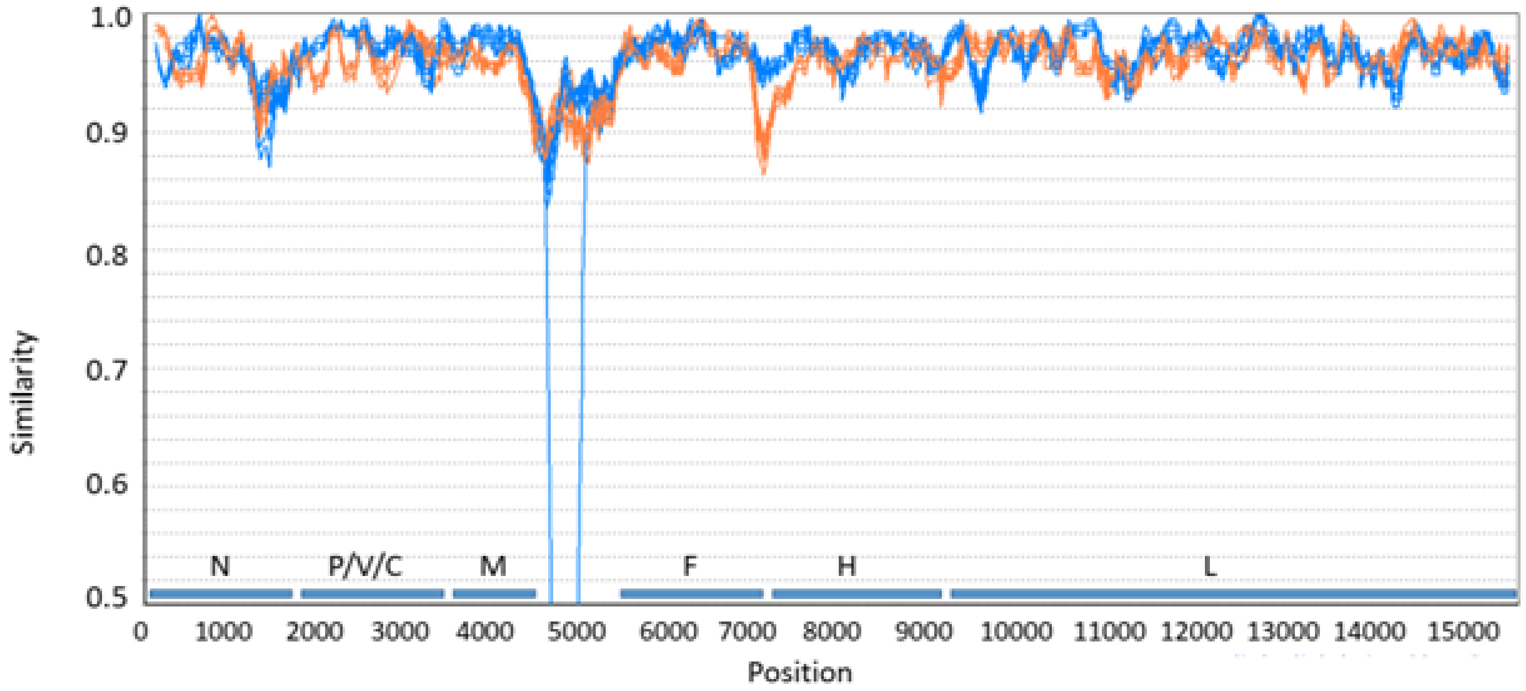

Supplementary Figure 1. Similarity plot of MeV WGS-t grouped by genotype with the open reading frames denoted. The similarity to the Edmonston reference sequence (GenBank AF266288, genotype A) over a 20 base step was plotted using a moving window of 200 bases. Orange: 20 B3 sequences; blue: 56 D8 sequences. The drop out of a single genotype D8 sequence in the M-F intergenic region is due to an insertion and deletion (indel) in the consensus sequence.

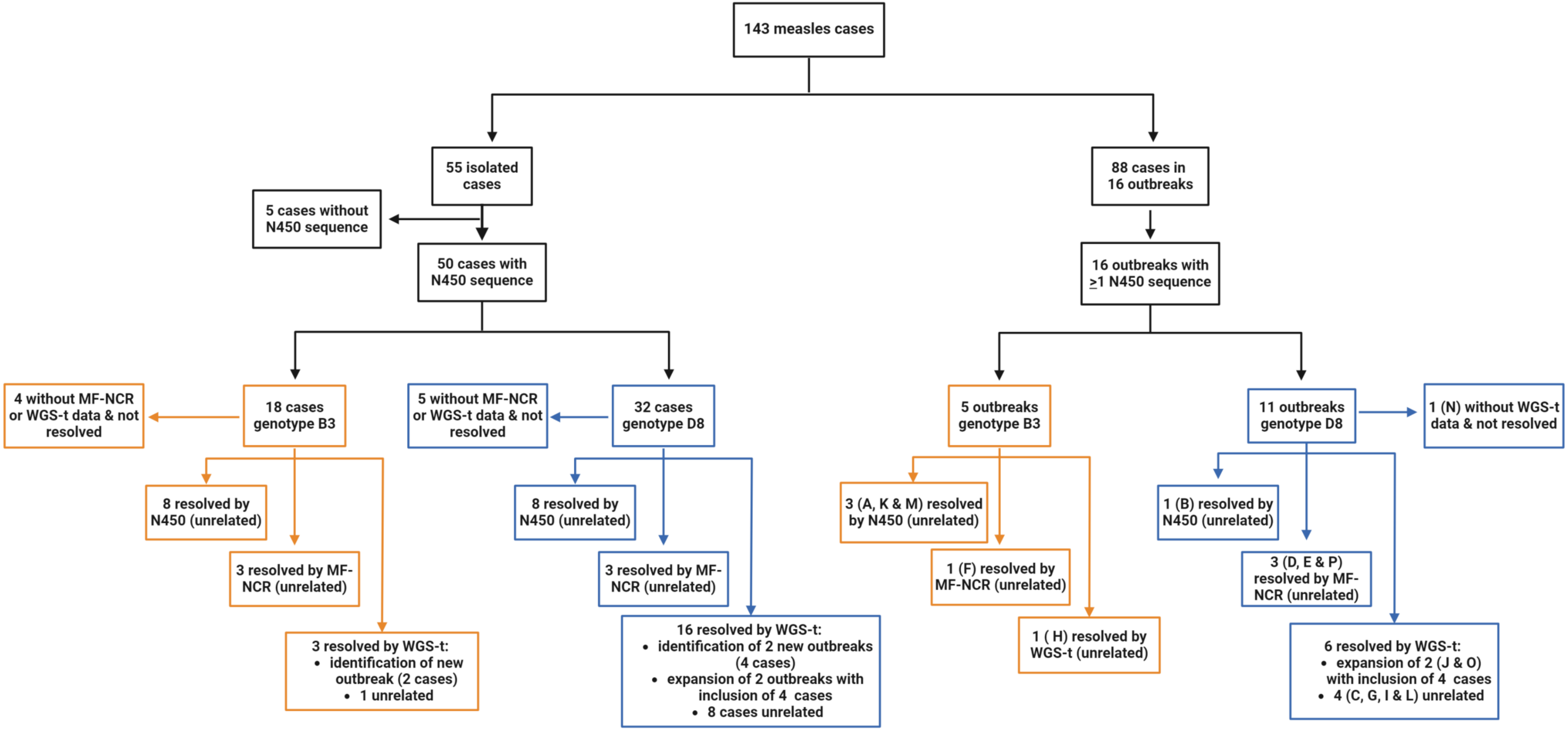

Supplementary Figure 2. Summary of outcome of sequence analysis for all measles cases. Cases were resolved with the sequence analysis in one of these ways: by the N450 sequence (phylogenetically or by the number of sequence differences and time from a putative common ancestor using the Poisson method described in Penedos et al 2022), by the MF-NCR sequence (phylogenetically) or by BEAST analysis of the WGS-t. Image prepared with Biorender.

Probability (α) that the relatedness of the pair can be excluded

DSId	Name		5	6	7	8	9	10	11	12	15	16	18	22	23	24	25	26	31	32
5306	MVs/Alberta.CAN/12.19_PHL	5																		
5306	MVs/British_Columbia.CAN/1.19_PHL	6	0																	
4274	MVs/British_Columbia.CAN/12.19_PHL	7	1	1																
5306	MVs/British_Columbia.CAN/14.19/2_ASIA	8	0	0	1															
5375	MVs/British_Columbia.CAN/25.18	9	5	5	4	5														
6018	MVs/British_Columbia.CAN/26.19_PHL	10	1	1	2	1	6													
6083	MVs/British_Columbia.CAN/29.19_PHL	11	2	2	3	2	7	3												
5654	MVs/British_Columbia.CAN/4.19_PHL	12	1	1	2	1	6	2	3											
5654	MVs/British_Columbia.CAN/7.19	15	1	1	2	1	6	2	3	0										
5793	MVs/British_Columbia.CAN/7.19/3_PHL	16	3	3	2	3	6	4	5	4	4									
6018	MVs/Manitoba.CAN/24.19_PHL	18	1	1	2	1	6	0	3	2	2	4								
5254	MVs/Ontario.CAN/14.18_UGD	22	5	5	4	5	6	6	7	6	6	6	6							
5306	MVs/Ontario.CAN/17.19_PHL	23	0	0	1	0	5	1	2	1	1	3	1	5						
5904	MVs/Ontario.CAN/18.19/2_PHL	24	1	1	2	1	6	2	3	2	2	4	2	6	1					
6218	MVs/Ontario.CAN/44.19_BGD	25	4	4	3	4	5	5	6	5	5	5	5	5	4	5				
5309	MVs/Ontario.CAN/8.19_PAK	26	4	4	3	4	3	5	6	5	5	5	5	5	4	5	4			
5230	MVs/Quebec.CAN/38.19	31	9	9	8	9	10	10	11	10	10	8	10	10	9	10	9	9		
5800	MVs/Quebec.CAN/6.19	32	11	11	10	11	12	12	13	12	12	10	12	12	11	12	11	11	6	

Number of nucleotides

- α = 0.99
- α = 0.97
- α = 0.95
- α = 0.90
- α = 0.85
- α = 0.80
- α < 0.80

Probabality (α) that the relatedness of the pair can be excluded

DSId	Name		7	8	9	10	11	13	14	15	16	17	18	19	20	23	49	50	51	52	53	54	55	60	61	62	63	65	66	67	68	77	78	80
5325	MVs/Alberta.CAN/22.18	7																																
5325	MVs/Alberta.CAN/22.18/2	8	0																															
4683	MVs/Alberta.CAN/27.18_UKR	9	1	1																														
5150	MVs/Alberta.CAN/4.18_IND	10	6	6	5																													
5877	MVs/Alberta.CAN/4.20	11	2	2	1	6																												
4683	MVs/British_Columbia.CAN/12.19/2	13	1	1	0	5	1																											
4683	MVs/British_Columbia.CAN/12.19/3	14	1	1	0	5	1	0																										
4683	MVs/British_Columbia.CAN/12.19/4	15	1	1	0	5	1	0	0																									
4683	MVs/British_Columbia.CAN/13.19	16	1	1	0	5	1	0	0	0																								
5840	MVs/British_Columbia.CAN/14.19_VNM	17	2	2	1	6	2	1	1	1	1																							
5601	MVs/British_Columbia.CAN/16.19_JPN/USA	18	2	2	1	6	2	1	1	1	1	2																						
5379	MVs/British_Columbia.CAN/25.18/2_IND	19	18	18	17	18	18	17	17	17	17	18	18																					
2283	MVs/British_Columbia.CAN/31.18_BRA	20	5	5	4	5	5	4	4	4	4	5	5	17																				
4683	MVs/British_Columbia.CAN/37.18	23	1	1	0	5	1	0	0	0	0	1	1	17	4																			
4801	MVs/Ontario.CAN/10.18_IND	49	6	6	5	6	6	5	5	5	5	6	6	18	5	5																		
4683	MVs/Ontario.CAN/12.19_PAK	50	1	1	0	5	1	0	0	0	0	1	1	17	4	0	5																	
4683	MVs/Ontario.CAN/13.19_GBR	51	1	1	0	5	1	0	0	0	0	1	1	17	4	0	5	0																
4683	MVs/Ontario.CAN/14.19_WPR	52	1	1	0	5	1	0	0	0	0	1	1	17	4	0	5	0	0															
5294	MVs/Ontario.CAN/18.18_IND	53	6	6	5	6	6	5	5	5	5	6	6	18	5	5	6	5	5	5														
4683	MVs/Ontario.CAN/18.19_USA	54	1	1	0	5	1	0	0	0	0	1	1	17	4	0	5	0	0	0	5													
4683	MVs/Ontario.CAN/19.19_FRA/DEU	55	1	1	0	5	1	0	0	0	0	1	1	17	4	0	5	0	0	0	5	0												
4683	MVs/Ontario.CAN/51.18_UKR	60	1	1	0	5	1	0	0	0	0	1	1	17	4	0	5	0	0	0	5	0	0											
4295	MVs/Ontario.CAN/6.18_IND	61	8	8	7	6	8	7	7	7	7	8	8	20	7	7	8	7	7	7	8	7	7	7										
5145	MVs/Ontario.CAN/7.18_IND	62	19	19	18	19	19	18	18	18	18	19	19	11	18	18	19	18	18	18	19	18	18	18	21									
4683	MVs/Ontario.CAN/7.19_THA/KHM	63	1	1	0	5	1	0	0	0	0	1	1	17	4	0	5	0	0	0	5	0	0	0	7	18								
4683	MVs/Quebec.CAN/12.19	65	1	1	0	5	1	0	0	0	0	1	1	17	4	0	5	0	0	0	5	0	0	0	7	18	0							
5823	MVs/Quebec.CAN/21.19_VNM	66	2	2	1	6	2	1	1	1	1	2	2	17	5	1	6	1	1	1	6	1	1	1	8	19	1	1						
5840	MVs/Quebec.CAN/24.19_VNM	67	2	2	1	6	2	1	1	1	1	0	2	18	5	1	6	1	1	1	6	1	1	1	8	19	1	1	2					
4683	MVs/Quebec.CAN/31.19_KHM	68	1	1	0	5	1	0	0	0	0	1	1	17	4	0	5	0	0	0	5	0	0	0	7	18	0	0	1	1				
4683	MVs/Quebec.CAN/4.19/2_UKR	77	1	1	0	5	1	0	0	0	0	1	1	17	4	0	5	0	0	0	5	0	0	0	7	18	0	0	1	1	0			
4683	MVs/Quebec.CAN/4.19_POL	78	1	1	0	5	1	0	0	0	0	1	1	17	4	0	5	0	0	0	5	0	0	0	7	18	0	0	1	1	0	0		
5970	MVs/Saskatchewan.CAN/22.19_IND	80	4	4	3	6	4	3	3	3	3	4	4	20	7	3	8	3	3	3	8	3	3	3	8	21	3	3	4	4	3	3	3	

Number of nucleotides

Supplementary Figure 3. Pairwise differences, in number of nucleotides (bottom of the grid), of single, isolated genotype B3 (top, n=18) and D8 (bottom, n=32) and the probability that the relatedness of the pair can be excluded (top of the grid). The probability is displayed as a heat map with the legend to the top right. The probability was determined by the number of sequence differences and time from a putative common ancestor (which for this analysis was set as an arbitrary common ancestor 2 weeks prior to the earlier case of the pair, based on an average incubation period of 14 days) using the Poisson method described in Penedos et al 2022.

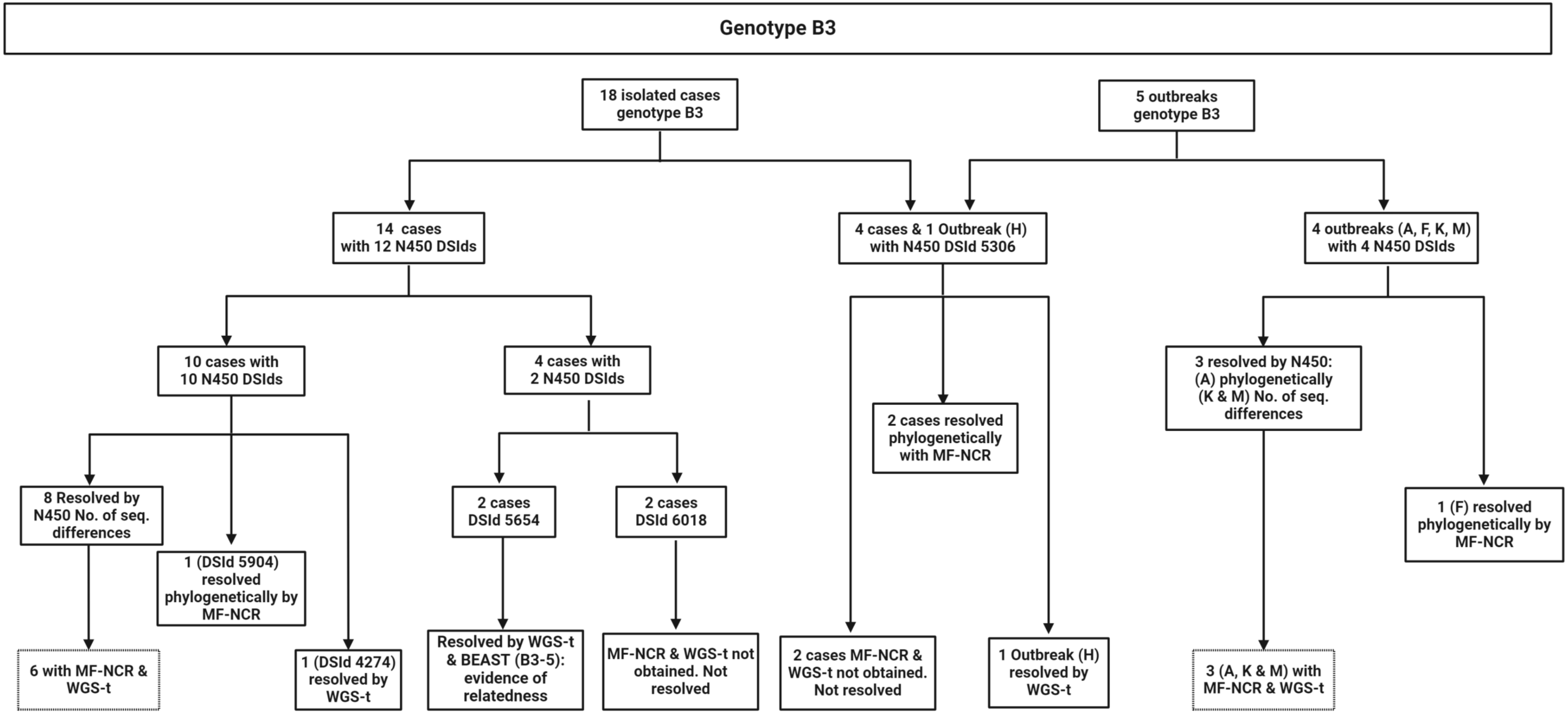

Supplementary Figure 4. Cases with genotype B3 sequences and outcome of sequence analysis. Cases were resolved with the sequence analysis in one of these ways: phylogenetically by the N450 sequence (bootstrap value ≥0.7), by the number of sequence differences and time from a putative common ancestor (which for this analysis was set as an arbitrary common ancestor 2 weeks prior to the earlier case of the pair, based on an average incubation period of 14 days) using the Poisson method described in Penedos et al 2022, phylogenetically by the MF-NCR sequence (bootstrap value >0.7) or by BEAST analysis of the WGS-t. Dotted lines around boxes indicate that this analysis was not needed to provide the molecular resolution. Image prepared with Biorender.

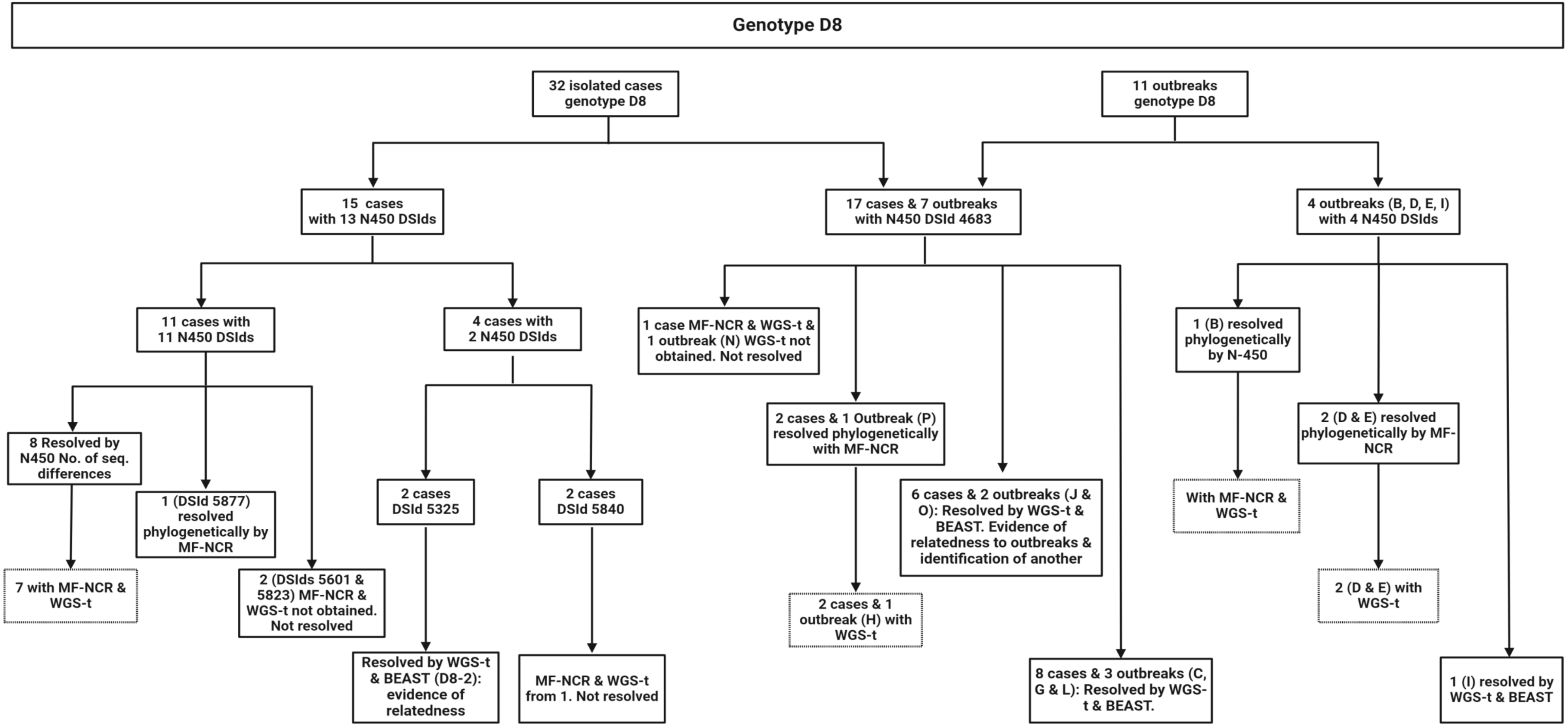

Supplementary Figure 5. Cases with genotype D8 sequences and outcome of sequence analysis. Cases were resolved with the sequence analysis in one of these ways: phylogenetically by the N450 sequence (bootstrap value ≥0.7), by the number of sequence differences and time from a putative common ancestor (which for this analysis was set as an arbitrary common ancestor 2 weeks prior to the earlier case of the pair, based on an average incubation period of 14 days) using the Poisson method described in Penedos et al 2022, phylogenetically by the MF-NCR sequence (bootstrap value >0.7) or by BEAST analysis of the WGS-t. Dotted lines around boxes indicate that this analysis was not needed to provide the molecular resolution. Image prepared with Biorender.

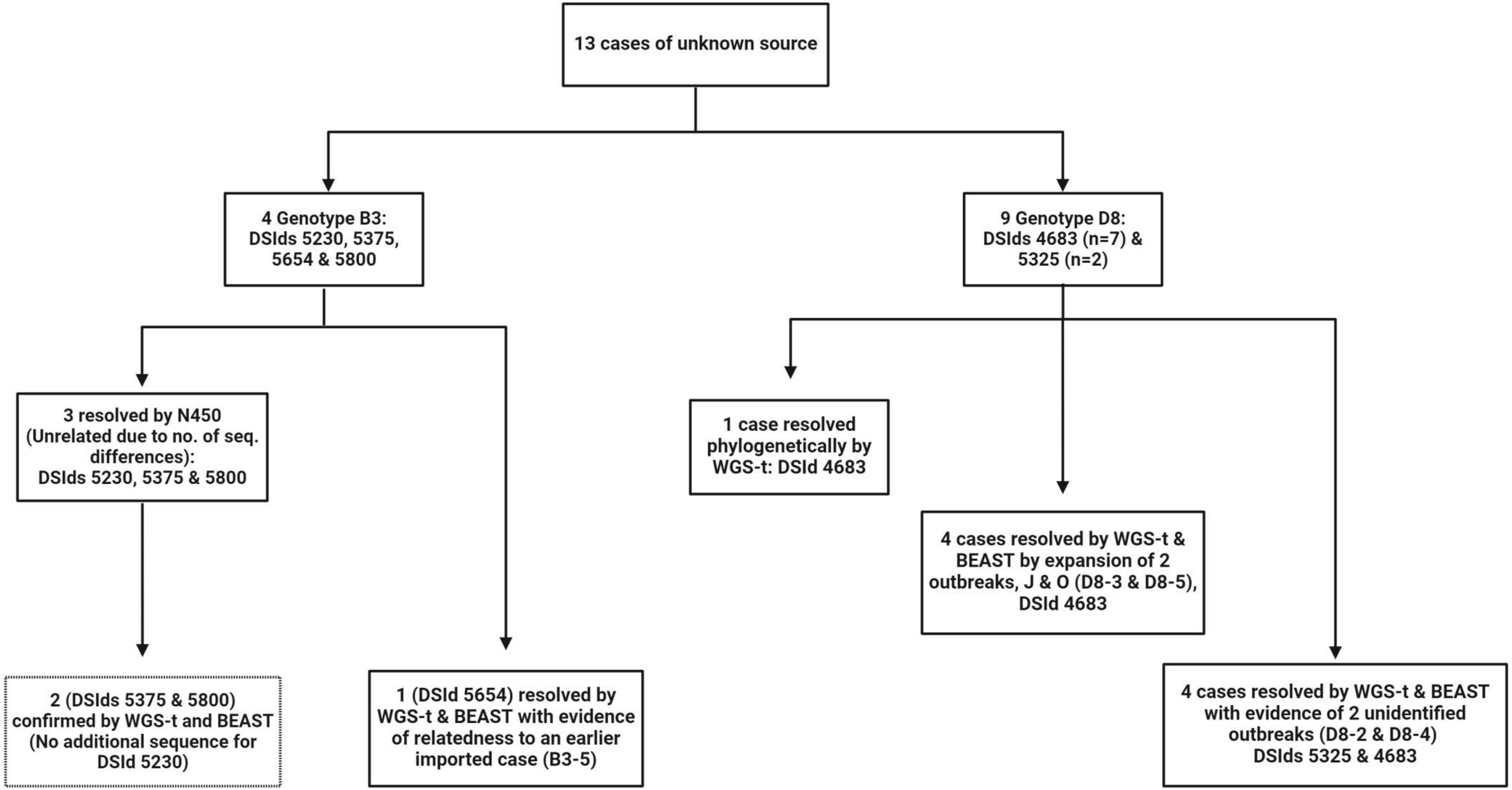

Supplementary Figure 6. Summary of outcome of sequence analysis for measles cases without an identified source. Cases were resolved with the sequence analysis in one of these ways: by the N450 sequence (phylogenetically or by the number of sequence differences and time from a putative common ancestor using the Poisson method described in Penedos et al 2022), by the MF-NCR sequence (phylogenetically) or by BEAST analysis of the WGS-t. Image prepared with Biorender.

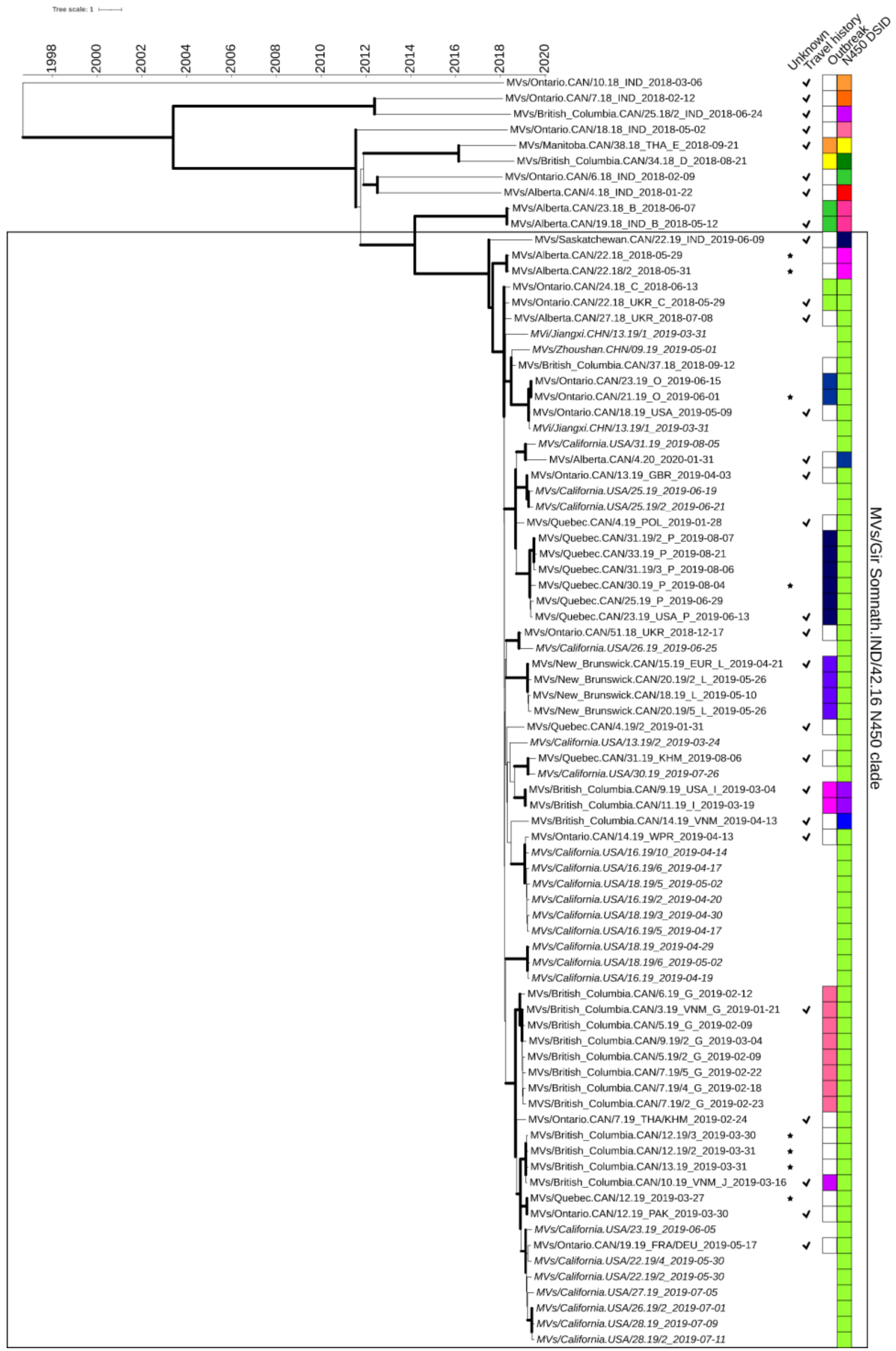

Supplementary Figure 7. Time scaled phylogenetic tree of MeV D8 WGS-t sequences with the additional of international sequences. Annotations (and legend) are as described in Figure 4. The time scale, in years, is at the top. International sequences are denoted by italic font. Thick branch edges correspond to posterior values ≥0.7. The box captures the sequences in the MVs/Gir Somnath.IND/42.16 N450 clade. High resolution, interactive versions are available at: https://itol.embl.de/shared/1hps8HAPCypnr

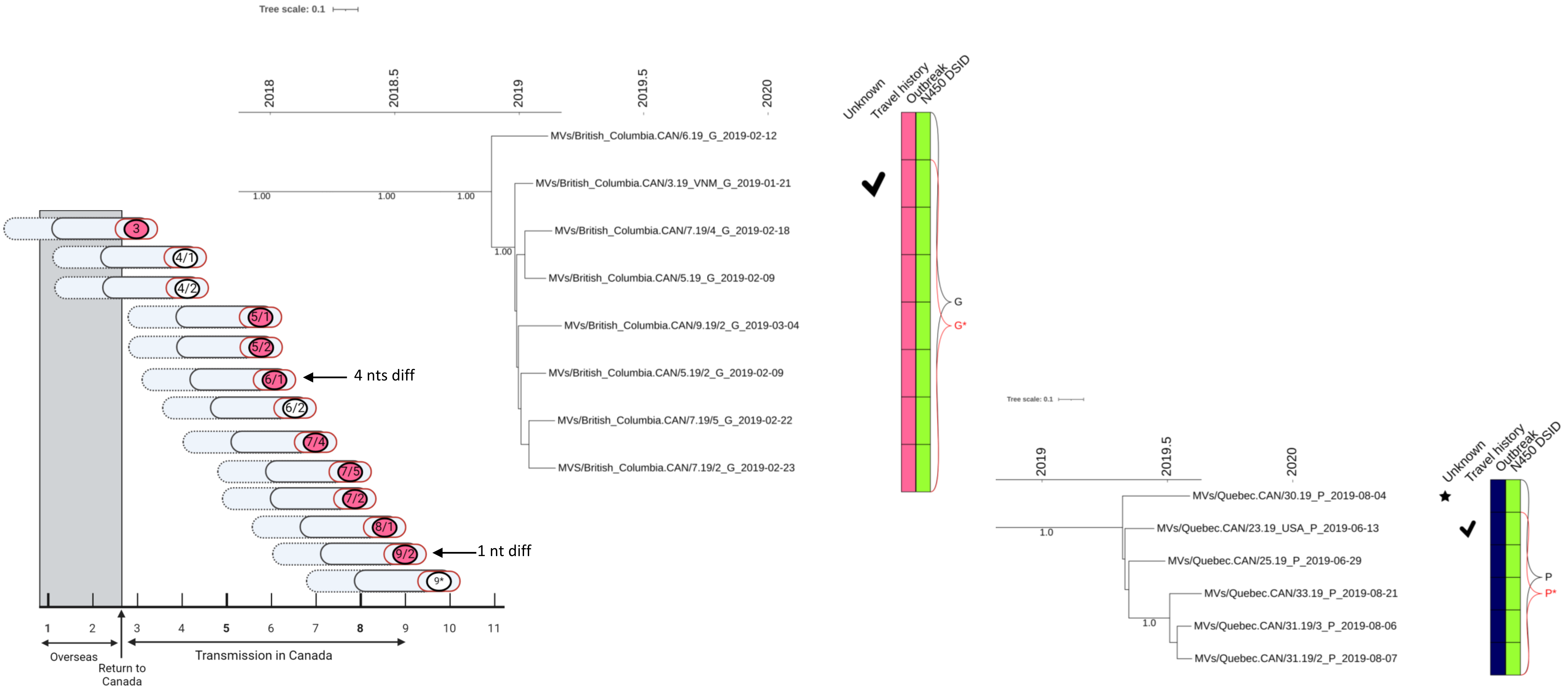

Supplementary Figure 8. Analysis of outbreaks G (left and centre) and P (right). The phylogenetic clades containing only the sequences of outbreak G (centre) and P (right) were pruned from the full time scaled WGS-t phylogenetic tree (Fig 6) to better see the clade structure and to identify the outlier sequences (MVs/British Columbia.CAN/6.19 for outbreak G and MVs/Quebec.CAN/30.19 for outbreak P). The time (horizontal) and vertical scales have been magnified, in comparison to Figure 6, and posterior values >0.7 are displayed as numbers on the branches rather than thick edges. **Left**: All cases attributed to outbreak G by the epidemiological investigation (n=13; Coulby et al 2021) are plotted out on a timeline based on the date of their rash onset (circles – empty and fuschia filled). Fuschia filled circles indicate cases with WGS-t. Numbers within the circles indicate the epidemiological week and can be used to correspond the timeline to the sequence (for example "3" is "MVs/British Columbia.CAN/3.19" and "7/4" is MVs/British Columbia.CAN/7.19/4", etc). Overlapping ovals represent expected infectious (red oval – 4 days before and after onset) and incubation times (black: average incubation time of 14 days; dotted: maximum expected incubation time of 21 days). Number of nucleotide (nt) differences from the first case ("3") is indicated by the text; if no text then they are identical.

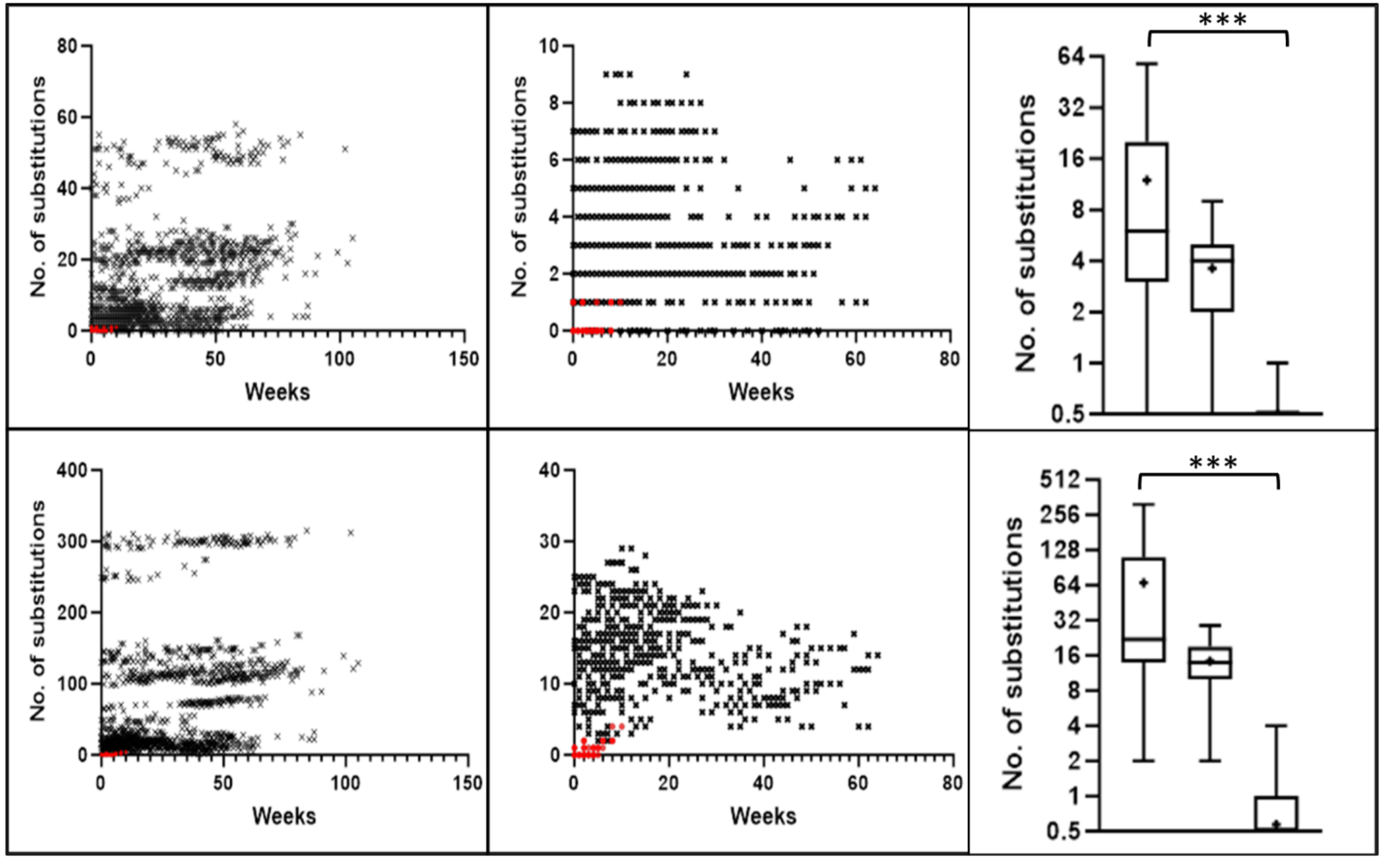

Supplementary Figure 9. Pairwise comparisons of MF-NCR (top) and WGS-t (bottom) sequences. Left and middle panels: number of nucleotide differences between pairs of sequences from cases confirmed to be related, either epidemiologically or through the WGS-t and BEAST analysis, (red circles) or not related (black x) plotted against the time, in weeks, between rash onset of the pairs of cases. Left panels: all pairs are included. Middle panels: only pairs with identical N450 sequences are included. Right panels: box and whisker plots of the number of nucleotide differences, log 2 scale, between pairs by related status (left: unrelated cases; middle: unrelated cases with identical N450 sequences; right: related cases). The minimum and maximum values are shown by the bars (whiskers) and the 25 to 75% quartiles are represented in the box with a line at the median value. The mean value is included as a plus sign. ***: Significant difference between mean number of differences between unrelated (left) and related (right) pairs of cases (Mann-Whitney test) with a p value <0.0001.
